# Mechanisms of reducing joint stiffness by blocking collagen fibrillogenesis in a rabbit model of posttraumatic arthrofibrosis

**DOI:** 10.1371/journal.pone.0257147

**Published:** 2021-09-07

**Authors:** Andrzej Steplewski, Jolanta Fertala, Ryan E. Tomlinson, Mark L. Wang, Allison Donahue, William V. Arnold, Michael Rivlin, Pedro K. Beredjiklian, Joseph A. Abboud, Surena Namdari, Andrzej Fertala

**Affiliations:** 1 Department of Orthopaedic Surgery, Sidney Kimmel Medical College, Thomas Jefferson University, Philadelphia, Pennsylvania, United States of America; 2 Rothman Institute of Orthopaedics, Thomas Jefferson University Hospital, Philadelphia, Pennsylvania, United States of America; 3 College of Medicine, Drexel University, Philadelphia, Pennsylvania, United States of America; University of Rochester, UNITED STATES

## Abstract

Posttraumatic fibrotic scarring is a significant medical problem that alters the proper functioning of injured tissues. Current methods to reduce posttraumatic fibrosis rely on anti-inflammatory and anti-proliferative agents with broad intracellular targets. As a result, their use is not fully effective and may cause unwanted side effects. Our group previously demonstrated that extracellular collagen fibrillogenesis is a valid and specific target to reduce collagen-rich scar buildup. Our previous studies showed that a rationally designed antibody that binds the C-terminal telopeptide of the α2(I) chain involved in the aggregation of collagen molecules limits fibril assembly *in vitro* and reduces scar formation *in vivo*. Here, we have utilized a clinically relevant arthrofibrosis model to study the broad mechanisms of the anti-scarring activity of this antibody. Moreover, we analyzed the effects of targeting collagen fibril formation on the quality of healed joint tissues, including the posterior capsule, patellar tendon, and subchondral bone. Our results show that blocking collagen fibrillogenesis not only reduces collagen content in the scar, but also accelerates the remodeling of healing tissues and changes the collagen fibrils’ cross-linking. In total, this study demonstrated that targeting collagen fibrillogenesis to limit arthrofibrosis affects neither the quality of healing of the joint tissues nor disturbs vital tissues and organs.

## Introduction

Balanced scar formation is crucial to natural wound healing [1]. However, when scarring is excessive, it negatively impacts the vital functions of the affected tissues. For example, fibrotic scars formed in muscles weaken them, and the scar tissue formed within and around injured joints causes arthrofibrosis [2, 3]. Arthrofibrosis, which can occur due to accidental trauma or surgery, stiffens the joints, restricts the ability to perform activities of daily living, and causes pain [4].

Because mature scars comprise insoluble collagen fibrils, they are incredibly stable and persist in the injury sites for months, years, or even permanently [5, 6]. Surgical removal of established scar tissue often exacerbates the problem, and the available pharmacological methods to reduce posttraumatic fibrosis are neither safe nor effective. For instance, intralesional injection of steroids to treat excessive skin scarring may promote infection, cause hyperpigmentation and skin atrophy [7]. Long-term use of topical mitomycin-c and 5-fluorouracil causes substantial ocular toxicity [8, 9]. Intraarticular injections of steroids may reduce arthrofibrosis, but their effect is usually short-term, and frequent steroid administration carries the risk of osteoporosis and infection [3, 10].

Consequently, optimal anti-scarring efforts should prevent scar formation rather than remove it after it has been firmly established. Our research group has identified collagen fibril formation as a valid anti-fibrotic target [11]. The fundamental premise for targeting collagen fibrillogenesis is that collagen fibrils are the main component of mature scars, hence limiting their formation attenuates unwanted effects of excessive scarring [11, 12].

To reduce the number of collagen fibrils formed in response to injury, we developed a monoclonal anti-collagen antibody (ACA) that targets the C-terminal telopeptide of the α2(I) chain (α2Ct), which is engaged in the aggregation of collagen monomers into fibrils [11, 13, 14]. Our studies with biologically and clinically relevant models of excessive scarring clearly demonstrated the ACA’s ability to reduce collagen fibrillogenesis and limit scar formation [11, 13–16].

Since the fibrotic scar and neotissue formed during the healing process have nearly the same composition, achieving the proper balance between reducing unwanted scarring and maintaining proper healing is challenging. As all anti-fibrotic agents alter the collagen-based matrix formation needed to repair injury sites, testing any novel anti-fibrotic approach requires defining its anti-fibrotic utility and the impact on intrinsic healing [17].

Here, we studied the impact of the ACA on healing connective tissues, including the posterior joint capsule (PC), subchondral bone (SB), and the patellar tendon (PT). While we understand the primary mechanism of the ACA-dependent inhibition of collagen fibril assembly, the overall secondary effects of this interference are not known. Furthermore, we analyzed the safety of using ACA by measuring its impact on vital cellular and biochemical parameters of the blood of treated animals and studying histological features of distant tissues and organs that were not directly targeted for treatment.

We demonstrated that direct blocking of collagen fibrillogenesis changes the dynamics of collagen metabolism and impacts the architecture and composition of the collagen-rich scar tissues formed in response to injury. Although applying the ACA following knee injury reduced the flexion contracture of healed joints, the antibody neither altered the healing of injured joint tissues nor negatively impacted distant tissues and organs.

Overall, our study demonstrated that targeting the collagen-rich scar tissue assembly offers an effective and safe method to reduce joint tissues’ posttraumatic fibrotic response. Moreover, discovering the secondary effects of ACA treatment suggests that blocking collagen fibrillogenesis has far-reaching beneficial consequences that enhance this antibody’s anti-fibrotic potential.

## Materials and methods

### Production of the ACA

The ACA was produced in Chinese Hamster Ovary (CHO) cells cultured in a bioreactor (New Brunswick BioFlo 115, Eppendorf, Enfield, CT USA) in a protein-free medium (CD CHO, ThermoFisher Sci., Waltham, MA USA) [14]. The conditioned medium containing the ACA was collected, then concentrated by ultrafiltration (ECO Cassette, Sartorius Stedim Biotech GmbH, Goettingen, Germany). Subsequently, the antibody was purified using the Protein-L resin (GenScript Biotech Corporation, Piscataway, NJ USA). Non-specific human IgG that served as a control antibody (CA) was purchased from a commercial supplier (Sigma-Aldrich, Allentown, PA USA). Each batch of CA was tested to exclude a possibility of its non-specific interaction with the α2Ct epitope.

### A rabbit model of arthrofibrosis

We employed an established rabbit model of posttraumatic joint contracture to study the effects of the ACA on the healing of the joint tissues, including the PC, SB, and PT [16, 18–21].

We utilized both male and female New Zealand White rabbits; 8 to 12 months old (Charles River Laboratories, Wilmington, MA USA). The animal studies were approved by the Institutional Animal Care and Use Committee (IACUC) of Thomas Jefferson University and the Animal Care and Use Review Office (ACURO) of a sponsoring agency.

Before applying general anesthesia, the rabbits received ketamine and xylazine. Subsequently, an analgesic was administered preoperatively and postoperatively to minimize the pain caused by surgery to the knee. At the end of experiments, the rabbits were euthanized by intravascular or intracardiac bolus overdose of a euthanasia agent containing barbiturate sodium pentobarbital.

All animal experiments complied with the Animal Research: Reporting of *In Vivo* Experiments (ARRIVE) guidelines. Table 1 provides detailed information about the rabbits used in our study.

**Table 1:** A summary of experimental groups and the number of rabbits in the ACA and control groups.

^a^Group (AB)	^b^Number of rabbits (M, F)	^c^Total AB delivered to an injured knee [mg]	^d^Experimental design (weeks of AB delivery + recovery period)	^e^Number of lost rabbits
12wk (+ACA)	8 (M), 7 (F)	4	8 + 4	1 (F)
12wk (+CA)	6 (M), 7 (F)	4	8 + 4	2 (F)
8wk (+ACA)	2 (M), 2 (F)	10	8 + 0	0
8wk (+CA)	2 (M), 2 (F)	10	8 + 0	0
8wk (+ACA)	2 (M), 2 (F)	60	8 + 0	0
8wk (+CA)	1 (M), 1 (F)	60	8 + 0	0

^a^AB, Antibody used.

^b^M, Males; F, Females.

^c^The amount of antibody received during the entire 8-week period.

^d^The 8-week period of antibody delivery and the 4-week period of recovery are indicated.

^e^All rabbits were lost while administering anesthesia (see below for autopsy results).

Since we were interested in posttraumatic arthrofibrosis, our model included damage to crucial elements of the knee joint, including the joint capsule, ligaments, tendons, cartilage, and subchondral bone. The model also included intraarticular bleeding. Necessary surgical procedures included the following steps: (i) An incision in the middle of the right knee was made starting proximal to the patella and extending distally to the tibial tubercle and down the tibia. (ii) A lateral parapatellar arthrotomy was made, and then the patella was dislocated medially. (iii) Following flexing the knee, the fat pad was released and retracted medially. (iv) The anterior crucial ligament (ACL) and the posterior crucial ligament (PCL) were transected, and the knee was hyperextended to 45° to disrupt the posterior capsule. (v) A full-thickness 3-mm osteochondral defect (OCD) was made in the medial femoral condyle at the weight-bearing apex. (vi) Beginning as far posteriorly in the notch as possible and exiting out through the lateral condyle, a 2-mm hole was made for a catheter. (vii) A custom-made silicone catheter with a fixed retention bead at one end was passed through the hole. While the retention bead secured the catheter inside the operated knee, the opposite end was connected with a subcutaneous pump placed so as not to affect the knee area (iPRECIO SMP-200, Primetech Co., Tokyo, Japan) [16]. (viii) The operated right leg was placed back in extension with the patellar tendon in a normal position. (ix) A full-thickness, 3 mm-wide, 10 mm-long central portion of PT was resected with a surgical blade [22]. The defect location was marked with 5–0 nonabsorbable nylon sutures. Creating this standard defect enabled a comparison of mechanical properties of the neotissue formed during the healing process. (x) The injured knee was fixed in the flexed position using a Kirschner (K)-wire, as described [16, 21]. (xi) Immediately after the surgery, the ACA was applied intraarticularly via a subcutaneous pump. (xii) Eight weeks later, the K-wires were removed, and the rabbits returned to their cages for 4 weeks. During the 4-week recovery period, the antibodies were not applied. In all rabbits, the left leg served as the uninjured control. S1 Fig in S1 File illustrates crucial surgical steps; for additional information please see our earlier reports [16, 21].

### Experimental and control groups

Here, we were interested in two aspects of the ACA-based treatment: (i) its long-term impact on the healing process, and (ii) safety.

To address the first aspect, we created a group, referred to as the 12wk group, that was maintained for 12 weeks following the initial surgery (Table 1 and S2 Fig in S1 File) [16, 21]. This group included 8 weeks of knee immobilization with the ACA delivery and 4 weeks of knee remobilization with no ACA delivery. The rabbits from the 12wk group received 0.5 mg/knee/week of the ACA, *i*.*e*., in total 4 mg/knee. This specific dosage was consistent with that used in our earlier studies on the ACA’s efficacy [16]. Based on these studies, we also calculated the number of rabbits needed for the 12wk group. Since the ACA decreases the amount of collagen (calculated as % of dry tissue mass) deposited in the PCs by 20% (with standard deviation SD = 16) compared to the control, we considered this reduction as the basis for the group size determination [16]. Consequently, 10 rabbits in each group have an 80% power to detect a difference between means of 21.26 with a significance level (alpha) of 0.05 (GraphPad StatMate version 2.00 for Windows, GraphPad Software, San Diego California USA).

Consistent with earlier studies, in the 12wk group we introduced the 4-week joint remobilization period to minimize the possible joint stiffening effect due to 8 weeks of immobilization [16, 20, 23]. Apart from the scarring due to the joint injury that was created surgically, the immobilization itself stiffens the joints due to, for example, rearrangement of collagen fibrils, lack of lubrication between collagen fibrils, and adaptive shortening of the joint capsule [24–26].

To fully utilize the 12wk group, we also employed it for the ACA safety assays as a part of dose escalation studies (see below).

To address the second aspect, we created the 8-week group to test the dose-dependent safety of the ACA. We applied a standard 4 + 4 design for the dose escalation study used commonly in phase I clinical tests [27].

In brief, a cohort of 4 rabbits (2 males and 2 females) is given the lowest dose ACA (Table 1). If none of the 4 rabbits exhibits a serious adverse effect, then the ACA is escalated to the next higher dose. If 1 or 2 of the first 4 rabbits exhibit a serious adverse effect, then another cohort of 4 rabbits is enrolled on the same dose. If <3 adverse effects are observed in the entire group of 8 rabbits, the ACA’s dose is again escalated to the higher dose. If more than 2 adverse effects (>2/4 or >2/8) are observed at any time, the dose is rendered unsafe.

The first 8wk group of 4 rabbits received 1.25 mg/knee/week of the ACA, *i*.*e*., in total, 10 mg/knee. The second group received 7.5 mg/knee/week or 60 mg/knee during 8 weeks of the ACA administration. To define the safety of the ACA, we assayed crucial biochemical and cellular parameters of the blood and analyzed histopathological features of tissues and organs. To fully utilize these 8wk groups, we also employed them to study the ACA’s action mechanisms and the scar tissue’s morphology, composition, and mechanical characteristics.

Since the primary goal of forming the 8wk groups was to study the ACA’s safety at different concentrations, organ histomorphology and blood parameters were analyzed separately for each subgroup. Other parameters were analyzed based on combined results from both 8wk subgroups.

### Analysis of blood parameters

We analyzed the ACA’s effects on cellular and biochemical parameters of the blood and crucial collagen metabolites. The samples were collected at the same time of the day to minimize the possible impact of circadian rhythm on blood parameters and collagen metabolites.

### Degradation of collagen molecules

Cross-linked collagen molecules that form the fibrillar architecture of mature tissues are relatively resistant to proteolytic digestion. In contrast, free collagen molecules that have not been incorporated into mature fibrils degrade rapidly [28]. Since ACA prevents free collagen molecules from aggregating into fibrils, we hypothesized that the degradation of these molecules produced in response to joint injury would increase with ACA administration.

To test this hypothesis, we analyzed the serum concentration of hydroxyproline (HP), a collagen-specific amino acid (S3 Fig in S1 File). We also analyzed fibril-derived fragments that included the C-terminal telopeptides cross-linked by trivalent pyridinoline and deoxypyridinoline (XL; S3 Fig in S1 File). While serum HP assays measure both the HP residues derived from degraded free collagen molecules and those from degraded collagen fibrils, cross-linked collagen I telopeptide fragments arise solely from degraded fibrils and are not influenced by the degradation of newly synthesized collagen molecules or by dietary collagen intake [29].

HP was assayed by a chemical method (Sigma-Aldrich), and XL was assayed with the enzyme-linked immunosorbent assay (ELISA; G-Biosciences, St. Louis, MO USA). All assays were done in triplicate.

### Biosynthesis of procollagen I

We also used ELISA to measure the serum concentration of the C propeptides (CP) of procollagen I as a marker of procollagen biosynthesis (MyBiosource, Inc., San Diego, CA USA) (S3 Fig in S1 File).

### Histological assays of collagen fibrils

Since the scarring of the PCs is the main contributor to the knee stiffness in the rabbit model of arthrofibrosis employed here, the PCs were dissected and analyzed, as described [16, 21, 30]. In brief, the histological sections of the PCs were stained with collagen-specific picrosirius red dye. By combining this staining technique with polarized-light microscopy, we could approximate the thickness, the organization, packing density, and to a certain degree, the collagen type-specific composition of the fibrils [21, 31–33]. Studies verified that as the thickness of fibers increases, their birefringence color changes under the polarized light microscope from green to yellow to orange to red, *i*.*e*., from shorter to longer wavelengths [31, 34–37].

Employing a polarizing microscope (Eclipse LV100POL, Nikon Inc., Melville, NY) and the NIS Elements software (Nikon Inc.), the following groups of the birefringence colors were defined in captured images: (i) green birefringence (GB, identifies thin, loosely packed fibrils), (ii) yellow birefringence (YB, identifies intermediate-thickness fibrils), and (iii) red birefringence (RB, identifies thick, tightly packed fibrils) [16, 21].

All fibrils present in captured viewing areas were analyzed, as described [21, 38]. In brief, the above birefringence colors were defined by applying the software’s “color threshold” function. Subsequently, the areas occupied by pixels corresponding to the defined colors were determined by the software. Considering the sum of all pixels to be 100%, the percentage of each color group in the analyzed samples was calculated. A minimum of three histological sections for control and three for injured PCs were analyzed per each rabbit. To ensure consistency in defining birefringence colors, the same threshold settings were applied to all images. Automated calculations of all fibrils in the viewing areas eliminated any potential bias. A one-way ANOVA was conducted to examine whether the percentage of subpopulations of collagen fibrils were different in the injured and uninjured capsules from the ACA-treated or the CA-treated groups (IBM SPSS Statistic for Windows, version 26, IBM Corp., Armonk, NY, USA).

### Fourier Transformed Infrared Spectroscopy (FTIR)-based assays of the collagen content and the collagen cross-links

FTIR spectroscopy of tissue samples provides information about their composition and spatial distribution of analyzed macromolecules [39]. Here, we used this method to compare the relative collagen content in the scar tissues formed in the PCs and OCDs of the ACA-treated and CA-treated rabbits. As references, we selected a protein-specific amide II peak and a peak corresponding to sulfated glycosaminoglycans (GAGs). Consequently, the sulfated GAGs peak/collagen peak ratios and amide II peak/collagen peak ratios were measured to determine the relative collagen content (S1 Table in S1 File) [40, 41]. Please note that the relation between the ratio values and collagen amount is negative.

We also analyzed the maturity of collagen cross-links by measuring the pyridinoline (PYR) peak/dehydro-dihydroxynorleucine (de-DHLNL) peak ratios [42, 43]. Hence, the maturity of collagen fibrils was defined as the relative content of the “mature” trivalent cross-links (PYR) vs. the “immature” divalent PYR precursor cross-links (de-DHLNL). Since the PYR/de-DHLNL ratio increases during the fibrotic process, assays of the cross-links provide a relevant parameter to evaluate the fibrotic status of collagen-rich deposits formed in the presence of the ACA [44–46].

All tissue samples were prepared as described earlier [47, 48]. In brief, paraffin-embedded 5-μm thick tissue sections were deposited on the MirrIR low-e microscope slides (Kevley Technologies, Chesterland, OH). An FTIR spectrometer (Spotlight 400, Perkin Elmer, Waltman, MA) was used to analyze the regions of interest (ROIs) corresponding to the PC collagen-rich areas. The measurements were done in the 4000 cm^-1^ to 748 cm^-1^ wavenumbers spectral range, at a pixel resolution of 50 μm, 8 scans per pixel, and a spectral resolution of 4 cm^-1^. Co-added spectra from scanned ROIs were generated with the Spectrum Image software (PerkinElmer, Inc.).

In all assays of the FTIR-derived spectra, overlapping peaks were deconvoluted and analyzed based on the second-order derivative spectra and pre-determined bell-type Gaussian peak fitting function using the OriginPro software (version 2021, OriginLab Corporation, Northampton, MA USA) (S4 Fig in S1 File) [49, 50].

### Assays of the flexion contracture

Assays of knee flexion contracture provide an overall readout for the ACA efficacy to reduce joint stiffness [16, 21]. These assays were done according to protocols developed for the model employed here using a custom-made instrument, (S5 Fig in S1 File) [16, 19, 21]. In brief, the injured or the contralateral uninjured leg was fixed in the instrument with the tibia and femur positioned at the right angle. This position was considered the starting point of zero degrees. Subsequently, applying the rate of loading set to 40°/min, an extension torque was applied to 0.2 Nm. Upon reaching the value of 0.2 Nm, the angle corresponding to the joint extension was recorded (S5 Fig in S1 File). The injured knee flexion contracture was then expressed as the uninjured control knee extension/the injured contralateral knee extension ratio [16, 19, 21]. Hence, relatively low ratio values indicated relatively mild flexion contracture, while large ratio values indicated relatively severe flexion contracture.

Our main interest was evaluating the 12wk group that included the recovery period [16, 21]. The extent of the joint stiffness measured after the recovery period reflects the extent of the PC scarring due to injury. In contrast, the stiffness measured right after 8 weeks of immobilization results from the scarring of injured PCs and changes occurring strictly due to joint immobilization.

### Assays of the quality of healed PTs

Because of the complexity of knee injury created here to cause posttraumatic joint stiffness, standardization of a model wound to study the ACA’s impact on the quality of neotissue formed during the healing process is challenging. To circumvent this problem, we generated a uniform defect in the PTs to evaluate the quality of healing by measuring the mechanical properties of neotissue.

Biomechanical assays of the neo-patellar tendons from the ACA-treated and the CA-treated rabbits were performed as described [51]. In brief, following the flexion contracture measurements and PC isolation, the remaining soft tissues were dissected, leaving a PT-tibia complex (S6 Fig in S1 File). The neo-patellar tendon formed during the healing phase was isolated from the bulk of the remaining medial and lateral portions of the PT. A corresponding central portion of a PT from an uninjured leg was also prepared as a control.

The tensile behavior of the patellar tendon complexes from the ACA-treated and CA-treated rabbits was analyzed, as described [51]. First, the patellar and tibial ends of the tendon complex were secured in custom fixtures (S6 Fig in S1 File). Next, tensile testing was performed using a material testing system (TA Instruments ElectroForce 3220 Series III) equipped with a 225 N load cell. The samples were pre-loaded (0.3 N), then conditioned for 10 cycles from 0 to 0.5 mm displacement at an elongation rate of 0.2 mm/s, similar to previous work [52, 53]. Finally, the tendons were loaded to failure using a displacement ramp with an elongation rate of 0.2 mm/s. Force and displacement were captured digitally, then converted to engineering stress and strain for analysis using custom software (GNU Octave). Outcomes included ultimate stress and Young’s modulus.

### Assays of OCD mineralization and scarring

**Fig 1:**
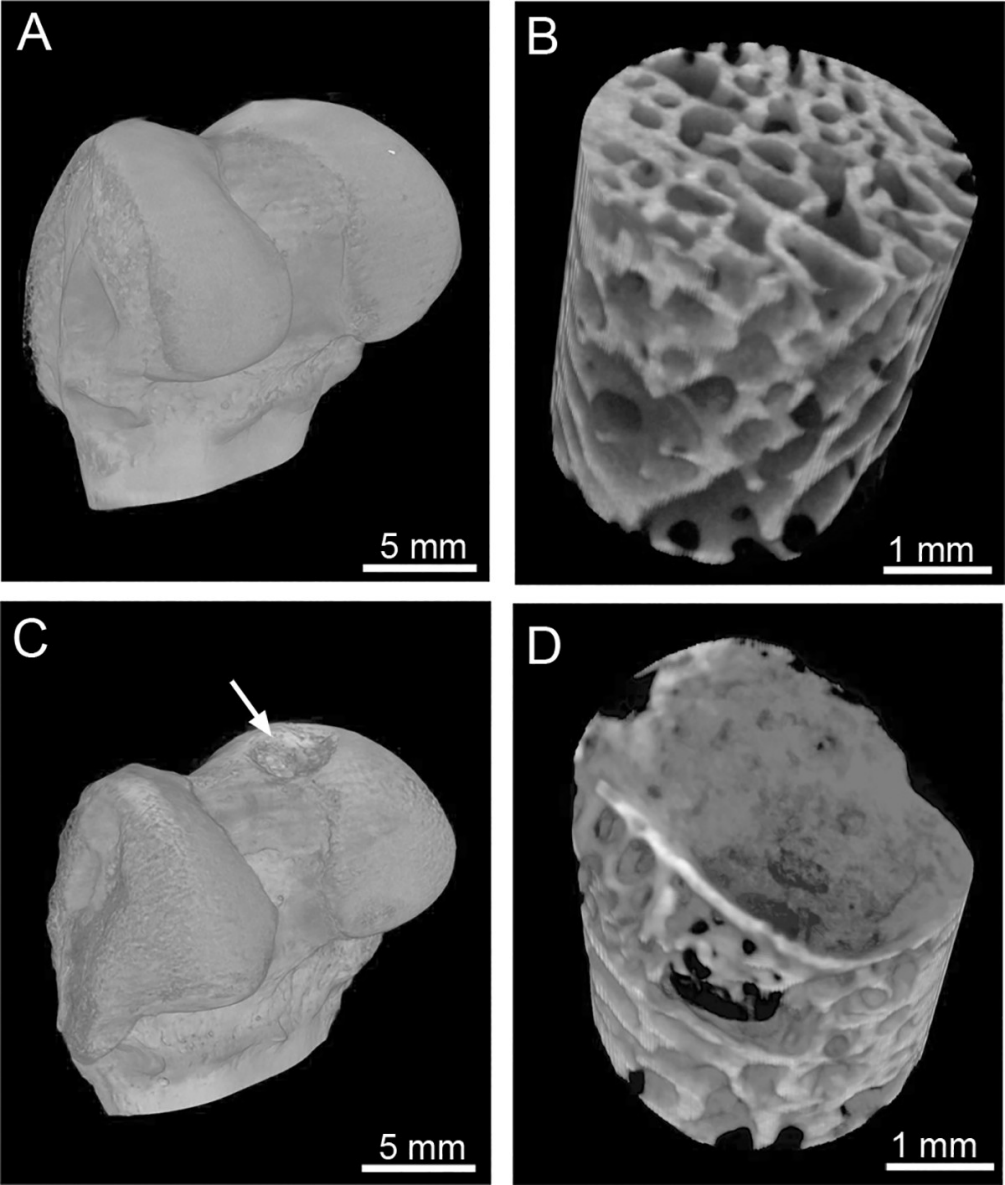
Representative computer tomography images of the uninjured (A) and injured (C, arrow) bones. Panels B (uninjured bone) and D (injured bone) indicate subchondral bone regions analyzed here to determine the extent of mineralization in the injured area (C&D).

We also studied the impact of the ACA on the healing of subchondral bone by measuring the calcification of the OCDs with a micro-computer tomography (μCT) scanner (SkyScan, Bruker Inc., Carteret, NJ, USA), as described [54]. Following reconstruction of μCT images, volumes of interest (VOIs) were selected in regions identified as the injury sites (Fig 1). Corresponding VOIs from contralateral uninjured legs were also analyzed. Our primary focus was on the Bv/Tv (percent of bone volume to the total analyzed volume) and the BMD (bone mineral density).

### Histology of new soft tissue formed within the OCDs

**Fig 2:**
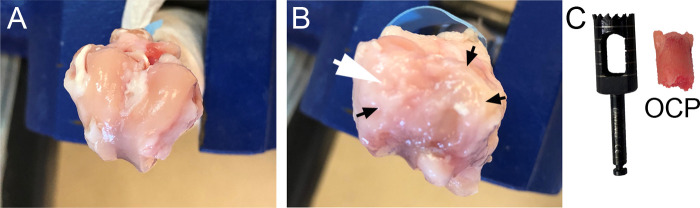
The appearance of the surface of uninjured (A) and injured (B) femoral condyles after twelve weeks of healing. The white arrow in B indicates the site of the osteochondral defect (OCD) made during the initial surgery. Please note the presence of the pannus-like tissue (black arrows) covering the femoral surface of the injured leg (B). C, An osteochondral plug (OCP) that encompasses the site of injury seen in panel B. A tool used to remove the plug is also indicated.

Following μCT, we isolated the tissue plugs encompassing the OCDs (Fig 2). The plugs were decalcified, processed for histology, then stained with hematoxylin and eosin (H&E) for general morphology and cellularity. The corresponding samples were also stained with picrosirius red to analyze the collagen-rich matrix of the newly formed tissue, as described above for the PC samples.

### Histology of tissues and organs

Following euthanasia, we collected samples of tissues and organs, including the brain, esophagus, thymus, aorta, heart, lung, liver, spleen, stomach, kidney, intestines, gonads, Achilles tendon, skeletal muscle, and the sciatic nerve. The samples were processed for histology, stained with H&E, and then observed for any abnormalities associated with the ACA treatment, including potential structural alterations and an influx of inflammatory cells.

### Data analysis

Due to the skewness of data, we applied the log transformation to minimize the skewness’s impact on statistical analyses. Consequently, results of assays of the serum markers of collagen metabolism, joint tissues’ mechanical properties, and FTIR-based analyses were expressed as geometric means (GM) and geometric mean ratios (GMR) instead of arithmetic means and mean differences.

Changes in the HP, CP and XL concentrations in the sera of the ACA-treated group were compared to the CA-treated control. Linear fitted curves were generated to determine the kinetics of changes from time-0 to week-8, a period of the ACA administration. Subsequently, the curves’ trajectories for the ACA and control groups were analyzed by comparing their slopes.

Furthermore, to analyze the HP, CP, and XL concentration changes across various time points within an analyzed group, we employed a within-group repeated-measures ANOVA with a Greenhouse-Geisser correction (IBM SPSS Statistics v. 26).

In all assays, statistical significance was defined as p ≤ 0.05.

For the flexion contracture, we formulated the superiority hypothesis. We expected that the injured limb in the ACA-treated group would show reduced flexion contracture compared to the injured limb in the control group:

$$H1:\;\mu_{1}[ACA:\;injured]\neq \mu_{0}[CTR:\;injured]$$

For the analyses, we estimated the mean difference between the ACA-treated and control groups (D = μ_1_ –μ_0_), along with the corresponding 2-sided 95% confidence limit and the p-value.

Also, it is often appropriate to test if a new experimental treatment is not unacceptably less efficacious than a control treatment. Hence, to study the effects of the ACA on the PT mechanical properties and calcification of the OCDs, we formulated the noninferiority hypothesis that the ACA treatment is not substantially worse than a control treatment. We computed the difference between the injured and contralateral non-injured limbs for each animal. We then compared the average of this difference for the ACA-treated animals and the CA-treated control.

Regardless of the treatment, we expected the injured limb’s values to be much lower than those for the uninjured leg. We aimed to show that this drop (from uninjured to injured limb) in the ACA-treated group is not meaningfully worse than in the control group (by a pre-specified margin):

$$H1:\;\mu_{1}[ACA:\;uninjured‐injured]<M\;*\;\mu_{0}[CTR:\;uninjured‐injured]\Rightarrow M\;*\;\mu_{0}–\mu_{1}>0$$

Here, M is the noninferiority margin. A relative margin of 20–33% (i.e., M = 1.20 to 1.33) is often appropriate since it is neither too broad (in which case it would be not very meaningful) nor too strict (in which case the study would require a considerable sample size). For example, a margin of 1.25 indicates that the worst-case scenario allowable for the ACA-treated group is an additional 25% beyond the decrease seen in the control group (always considering the difference between uninjured and injured limb).

Taking Young’s modulus (MPa) of the PT as an example derived from a published similar rabbit model, if the mean in the control group is 800 (MPa) for the uninjured PT and 300 (MPa) for the injured PT, the mean difference in this group is 500 [22]. A margin of 25% means that we want to establish that the corresponding actual mean difference between uninjured and injured PTs in the treated group is no bigger than 625 (1.25*500), *e*.*g*., the mean of 800 in the uninjured PT vs. 175 or larger in the injured PT (and hence a difference of 625 or less).

For the analyses, we computed the quantity D = M*μ_0_ –μ_1_, along with the corresponding 1-sided (upper) 95% confidence limit and 1-tailed p-value (the non-inferiority hypothesis is inherently 1-sided).

## Results

### A rabbit model of arthrofibrosis

Out of 42 operated rabbits in this study, two females (referred to as #1 and #2) died during pre-operative anesthesia (Table 1). The third female (referred to as #3) broke the K-wire ten days after the first surgery and subsequently died during the application of anesthesia for the K-wire replacement surgery. Also, rabbit #3 demonstrated somewhat agitated behavior with signs of self-mutilation.

Histopathological evaluation of tissues and organs collected from these female rabbits was performed by IDEXX BioAnalytics (Columbia, MO USA). Some underlying pathologies were noted. Female #1: heart: multifocal myocardial degeneration and fibrosis in the ventricles, hypertrophy of the tunica media and reduced lumen diameter in one small cardiac artery; kidney: mild hyperplasia of the collecting duct epithelium; liver: small yellow-green nodules cytoplasmic granules in periportal hepatocytes. Female#2: lung: mildly underinflated, mild scattered alveolar macrophages, subpleural accumulation of heterophils with alveolar macrophages. Female #3: heart: degradation of cardiomyocytes with mild fibrosis and scattered mononuclear cell infiltrates; lung: mild to moderate heterophils within alveolar capillaries and larger vasculature. We observed no other physical or behavioral problems with the rest of the rabbits (Table 1); all of them were included in all assays.

### Blood collection and analysis

Blood samples were collected at defined time points, and their cellular and biochemical parameters were analyzed. Overall, the blood parameters values remained within the physiological ranges defined for the rabbits throughout the entire experiment (S7–S10 Figs in S1 File) [55].

### Trajectories of changes in the concentration of the serum markers of collagen metabolism

We analyzed changes in the serum concentration of crucial collagen metabolites measured from the time of injury until the end of the 8-week antibody-application period. The serum concentration of HP, CP, and XL at specific time points were analyzed by plotting the data, fitting linear curves, and comparing their slopes for the ACA group and the CA group (Table 2). While the steep slopes (high numerical values) indicated relatively fast changes in measured parameters, more shallow slopes (low numerical values) signified relatively slow changes. Data indicate that the dynamics of changes in the HP concentration and the CP concentration were similar in the ACA and control groups. In contrast, the rate of increase in the XL concentration in the ACA group was significantly faster than in the control group (Table 2).

**Table 2:** A summary of differences in the trajectories of changes in serum hydroxyproline (HP), the C-terminal propeptide of procollagen I (CP), and cross-linked telopeptides (XL) measured between the ACA group and the CA group during the 8-week treatment period.

Parameter	Group	^a^GMR/wk (slope of lineary fitted curves)	The effect of ACA treatment (ACA/CA) with confidence levels and *p* values	
**HP (μg/ml)**	CA	1.01	ACA/CA	1.00
	ACA	1.01	95% CI^b^	(0.98,1.02)
			*p* = 0.892	
**CP (ng/ml)**	CA	1.04	ACA/CA	1.01
	ACA	1.05	95% CI	(0.97,1.05)
			*p* = 0.559	
**XL (ng/ml)**	CA	1.10	ACA/CA	1.28
	ACA	1.41	95% CI	(1.12,1.46)
			*p* = 0.001	

^a^GMR/wk, geometric mean ratios.

^b^CI, confidence interval.

Please note that GMR/wk presented in Table 2 represent the geometric mean ratio per week, i.e., the estimated GM today divided by the estimated GM a week ago. For instance, for XL (Table 2) measured for the 0-8-week period, that is 1.10 for CA and 1.41 for the ACA group, i.e., every week, XL increased on average by 10% and 41%, respectively. The effect of ACA treatment is 1.41/1.1 = 1.28, i.e., the ACA-associated increase per week is ~28% larger than the increase per week in the control group.

### Changes in the serum collagen markers across various time points

**Fig 3:**
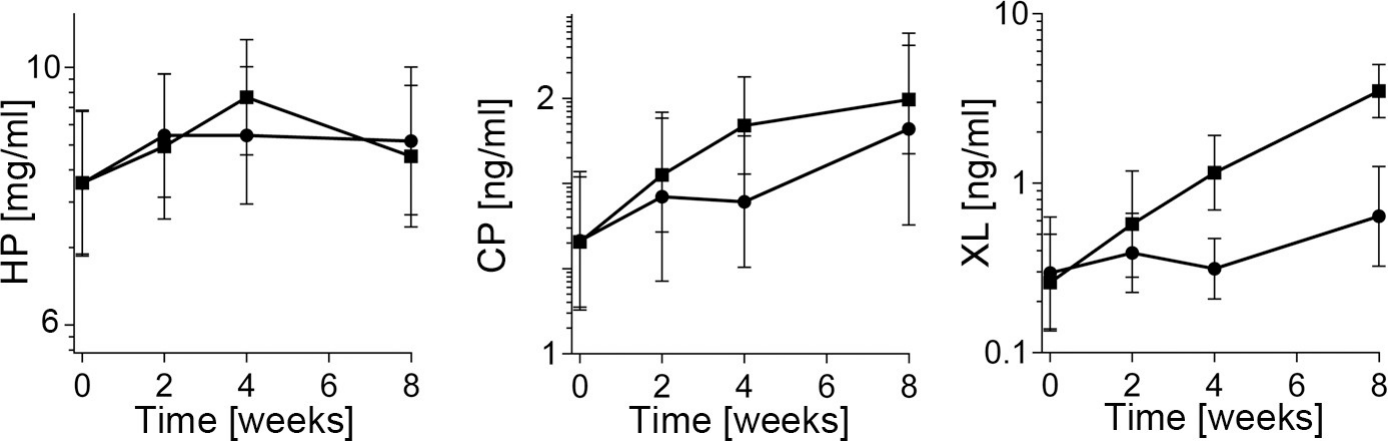
A graphic representation of the changes in the concentration of hydroxyproline (HP), the C-terminal propeptide (CP), and cross-linked telopeptides (XL) at indicated time points; the square symbols (■) represent the ACA-treated group, and the circle symbols (●) represent the control group. The geometric means (GMs) of analyzed parameters and 95% confidence intervals are presented.

To analyze changes in HP, CP, and XL within the ACA group and within the CA group across specific time intervals, we utilized the repeated-measures ANOVA (Fig 3, Table 3). As demonstrated in Table 3, there were no significant changes in the CA-treated rabbits. In contrast, statistically significant changes were observed in all analyzed parameters in the ACA-treated animals (Table 3).

**Table 3:** Significance of changes in serum concentrations of hydroxyproline (HP), the C-terminal propeptide of procollagen I (CP), and cross-linked telopeptides (XL). These changes were measured within the ACA group and within the CA group across various time points.

Marker	Treatment	Test within-subject effects (Greenhouse-Geisser)	Statistical significance of changes between specific time points (weeks)					
			0–2	0–4	0–8	2–4	2–8	4–8
**HP**	CA	*F*(2.218, 28.837) = 3.085, *p* = 0.056	n.s	n.s	n.s	n.s	n.s	n.s
	ACA	*F*(1.840, 36.808) = 4.734, *p* = 0.017	n.s	*p* = 0.009	n.s	n.s	n.s	*p* = 0.038
**CP**	CA	*F*(1.436, 20.102) = 3.123, *p* = 0.08	n.s	n.s	n.s	n.s	n.s	n.s
	ACA	*F*(2.261, 42.966) = 12.439, *p* < 0.005	*p* = 0.039	*p* = 0.006	*p* = 0.002	*p* = 0.01	n.s	n.s
**XL**	CA	*F*(1.546, 13.914) = 1.844, *p* = 0.198	n.s	n.s	n.s	n.s	n.s	n.s
	ACA	*F*(2.148, 27.929) = 18.669, *p* < 0.005	n.s	*p* = 0.007	*p* < 0.005	n.s	*p* = 0.007	n.s

n.s, not significant.

### Collagen matrix formed in the PCs

**Fig 4:**
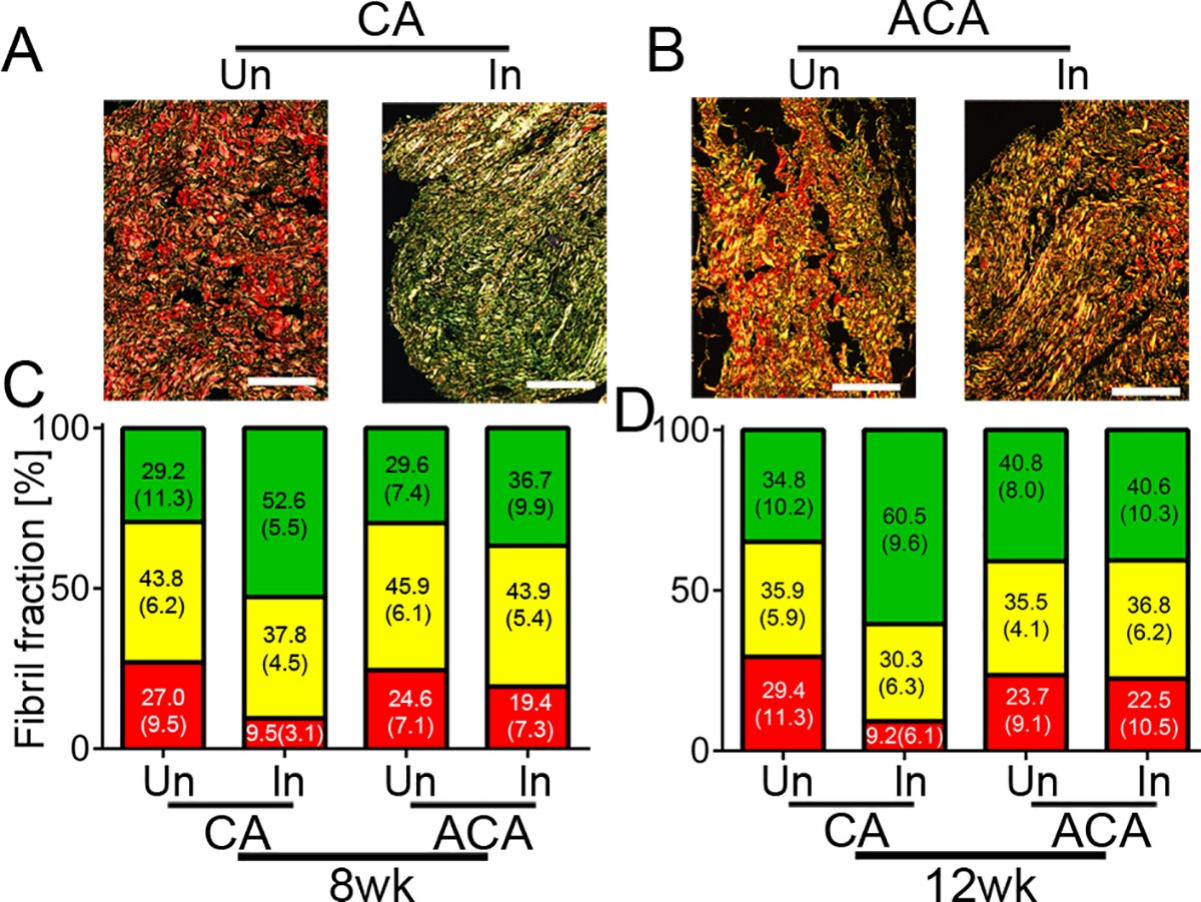
Histological quantification of various populations of picrosirius-stained collagen fibrils formed within injured posterior capsules (PCs). A, Samples of the PCs from uninjured (Un) and injured (In) knee joints of rabbits treated with control antibody (CA). B, Samples of the PCs from uninjured (Un) and injured (In) knee joints of the rabbits treated with the ACA. C&D, Corresponding graphs depict the percentages, with standard deviations in parentheses, of the green birefringence (GB), yellow birefringence (YB), and red birefringence (RB) subpopulations of collagen fibrils observed with the use of a polarized light microscope in the 8wk and 12wk groups. Bars = 100 μm.

We also examined the effects of the ACA and CA on the percentages of the green-birefringence (GB), the yellow-birefringence (Y(B, and the red-birefringence (RB) subpopulations of collagen fibrils present in the uninjured and injured PCs (Fig 4). Table 4 summarizes one-way ANOVA tests to determine the statistical significance of observed differences between analyzed groups.

**Table 4:** Analysis of differences between specific groups of collagen fibrils, defined by specific birefringence, observed in uninjured and injured posterior capsules (PCs) isolated from the ACA-treated or control rabbits.

Group	Treatment	Fibril subpopulation		
		^a^GB	^a^YB	^a^RB
**8wk**	CA	*F*(1, 10) = 20.7, *p =* 0.001	*F*(1, 10) = 3.6, *p =* 0.088	*F*(1, 10) = 18.1, *p =* 0.002
	ACA	*F*(1, 14) = 2.6, *p* = 0.129	*F*(1, 14) = 0.447, *p* = 0.514	*F*(1, 14) = 2.05, *p* = 0.175
**12wk**	CA	*F*(1, 20) = 37.0, *p* < 0.0005	*F*(1, 20) = 4.5, *p* = 0.046	*F*(1, 20) = 27.1, *p* < 0.0005
	ACA	*F*(1, 26) = 0.004, *p =* 0.949	*F*(1, 26) = 0.459, *p* = 0.504	*F*(1, 26) = 0.091, *p* = 0.765

^a^GB, green birefringence (represents thin, loosely-packed fibrils); YB, yellow birefringence (represents intermediate-diameter fibrils); RB, red birefringence (represents thick, well-packed fibrils).

### Collagen matrix formed in the OCDs

**Fig 5:**
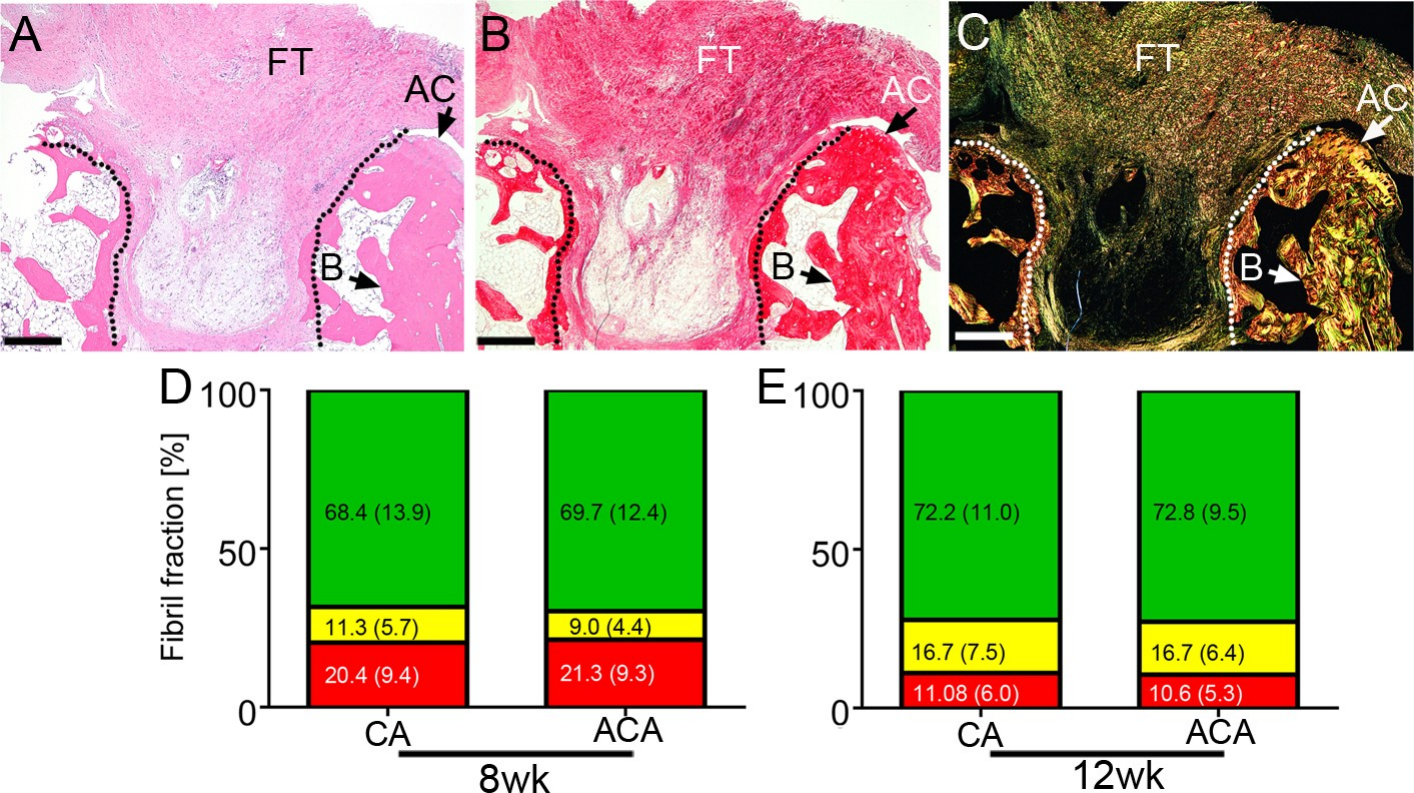
A histological assay of the pannus-like scar tissue formed in an osteochondral defect (delineated). The site of injury was stained with H&E (A) and with collagen-specific picrosirius red (B&C). The picrosirius red-stained samples were observed in normal light (B) and polarized light (C). Fibrotic tissue (FT), bone (B), and fragments of the articular cartilage (AC) are indicated. D&E, A summary of measurement of the percentages of the green birefringence (GB) fibrils, yellow birefringence (YB) fibrils, and red birefringence (RB) fibrils formed in the CA-treated and the ACA-treated rabbits. Corresponding segments of the bars include the means and standard deviations (in parentheses). Data for the 8wk and the 12wk groups are presented. Bars = 1 mm.

We also analyzed the ACA impact on the fibrillar composition of the pannus-like tissue formed around and within OCDs (Fig 5). As adequate controls from the contralateral joints’ uninjured sites are innately unavailable, we compared pannus-like tissues formed in the ACA and control groups. As indicated in Table 5, there were no statistically significant differences in the percentages of specific populations of fibrils present in the ACA-treated and CA-treated groups.

**Table 5:** Significance of differences between the content of specific groups of collagen fibrils defined by the green (GB), yellow (YB), and red (RB) birefringence. The fibrils were observed in the osteochondral defects in the CA-treated and the ACA-treated rabbits.

Group	Fibril subpopulation		
	^a^GB	^a^YB	^a^RB
**8wk**	*F*(1, 48) = 0.12, *p* = 0.731	*F*(1, 48) = 2.3, *p* = 0.137.	*F*(1, 48) = 0.117, *p* = 0.733
**12wk**	*F*(1, 142) = 0.102, *p* = 0.75	*F*(1, 142) = 0.001, *p* = 0.979	*F*(1, 142) = 0.3, *p* = 0.585

^a^GB, green birefringence (represents thin, loosely-packed fibrils); YB, yellow birefringence (represents intermediate-diameter fibrils); RB, red birefringence (represents thick, well-packed fibrils).

### FTIR assays of the collagen content in the PC and OCD scars

**Fig 6:**
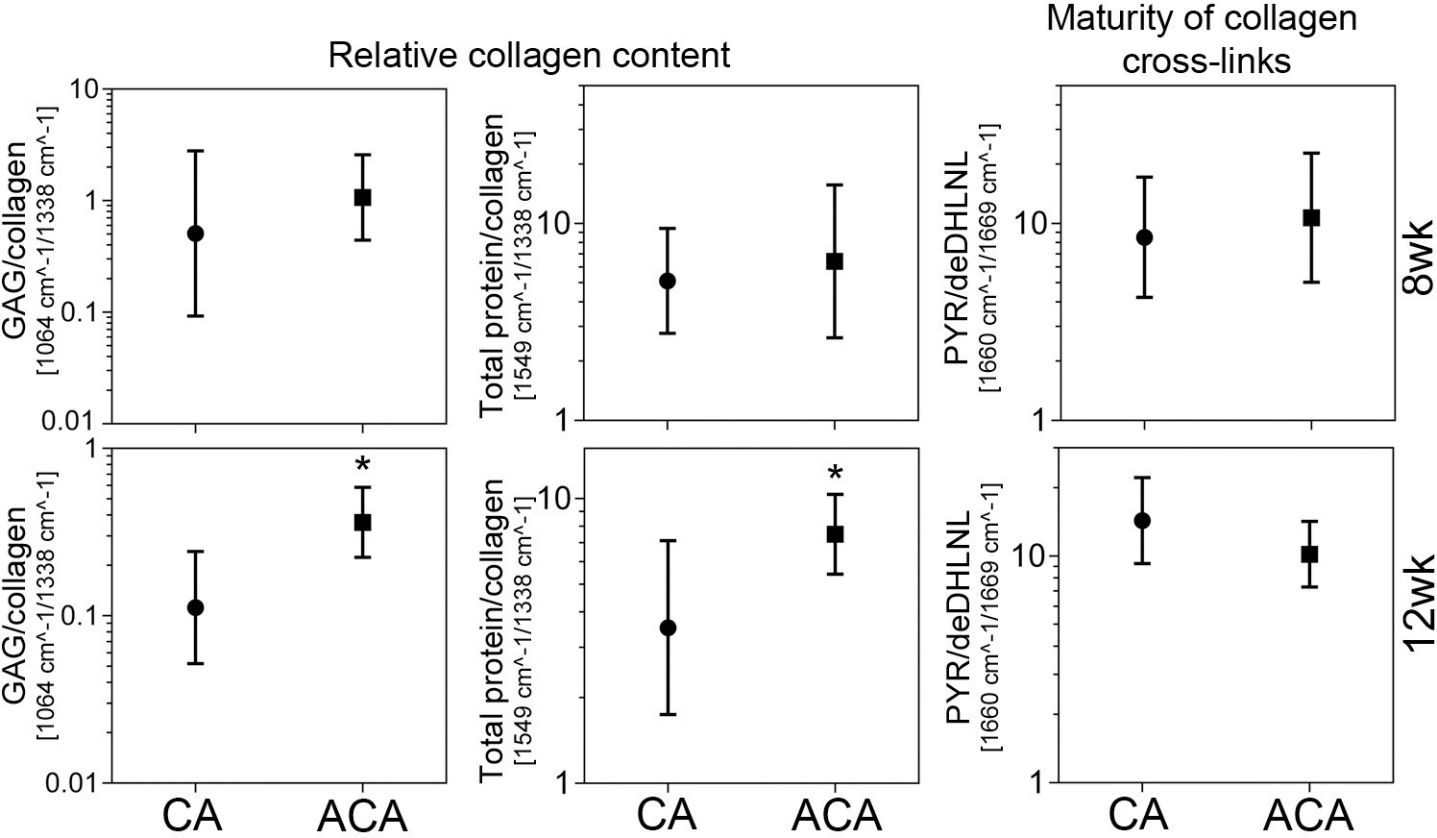
A graphic representation of the FTIR-based assays of the relative amount of collagen and the maturity of collagen cross-links in the scar tissue formed within injured posterior capsules of control (CA) and the ACA-treated rabbits. Results for the 8wk and the 12wk groups are presented. The graphs show geometric means (GMs) with 95% confidence intervals. Asterisks indicate groups with statistically significant differences of the means. The graph shows the relative collagen content calculated as the sulfated glycosaminoglycans (GAGs)/collagen and the total protein/collagen ratios. Consequently, the smaller the ratios, the higher relative collagen content. The ratios were calculated based on the areas of the spectral peak corresponding to the sulphated GAGs (centered around 1064 cm^-1^) and the spectral peak corresponding to collagen (centered around 1338 cm^-1^). To corroborate these measurements, the relative amount of collagen was also calculated based on the ratios of the spectral peak corresponding to the total proteins, represented by the amide II peak (centered around 1549 cm^-1^) and the spectral peak corresponding to collagen. The maturity of collagen cross-links was expressed as the ratio of the area of the spectral peak corresponding to the mature trivalent PYR cross-link (centered around 1660 cm^-1^) and immature divalent deDHLNL cross-link (centered around 1690 cm^-1^). Hence, the higher the PYR/deDHLNL ratio the more mature collagen cross-links. Also, see S1 Table in S1 File.

**Fig 7:**
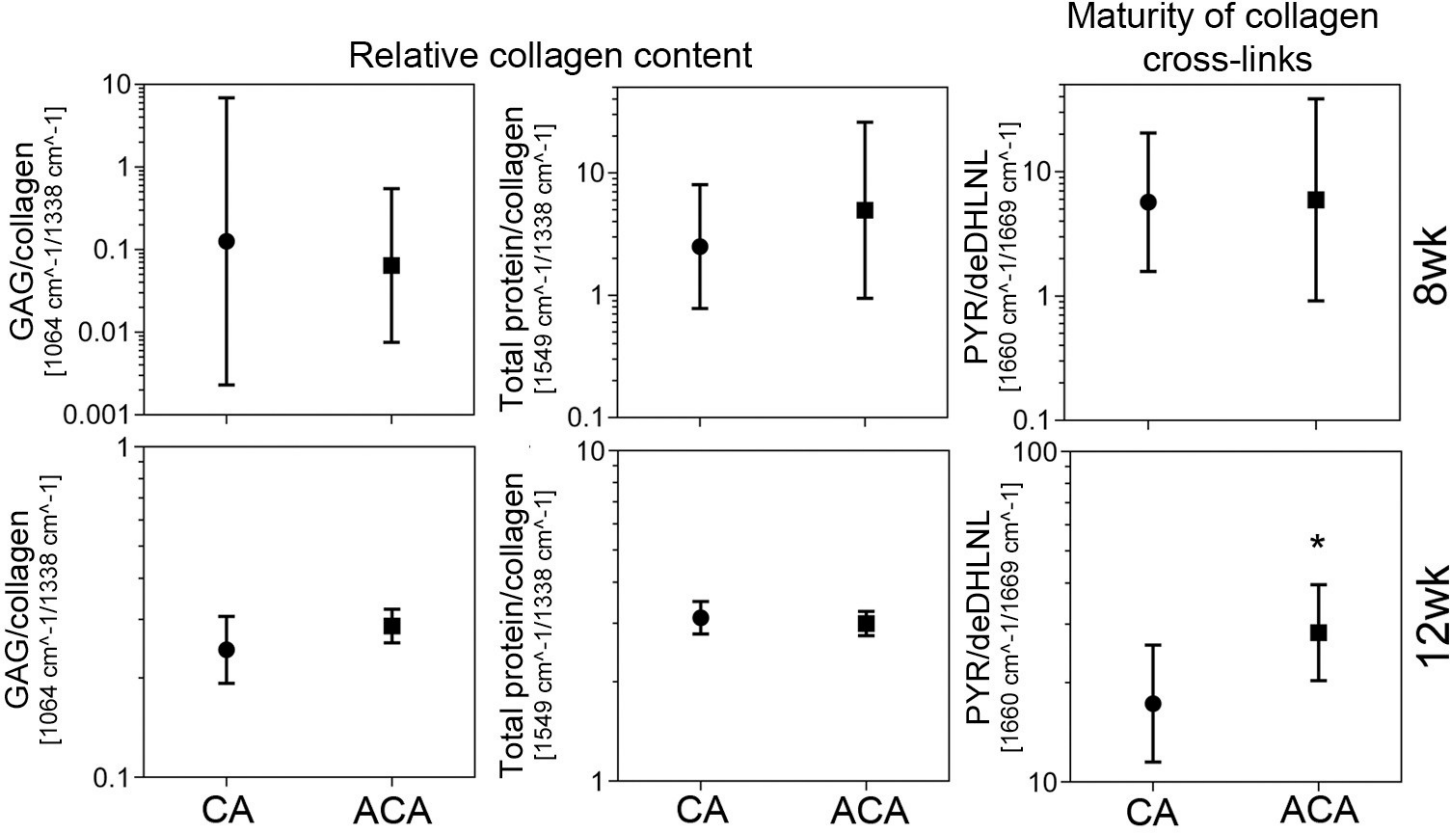
A graphic representation of the FTIR-based assays of the relative amount of collagen and the maturity of collagen cross-links in the scar tissue formed in the OCDs of the CA-treated and the ACA-treated rabbits. Results for the 8wk and the 12wk groups are presented. The graphs present GMs with 95% confidence intervals. Asterisks indicate groups with statistically significant differences of means. The graph shows the relative collagen content calculated as the GAG/collagen and the total protein/collagen ratios. Hence, the smaller the ratios, the higher relative collagen content. The ratios were calculated based on the areas of the spectral peak corresponding to the sulphated GAGs (centered around 1064 cm^-1^) and the spectral peak corresponding to collagen (centered around 1338 cm^-1^). To corroborate these measurements, the relative amount of collagen was also calculated based on the ratios of the spectral peak corresponding to the total proteins, represented by the amide II peak (centered around 1549 cm^-1^) and the spectral peak corresponding to collagen. The maturity of collagen cross-links was expressed as the ratio of the area of the spectral peak corresponding to the mature trivalent PYR cross-link (centered around 1660 cm^-1^) and immature divalent deDHLNL cross-link (centered around 1690 cm^-1^). Thus, the higher the PYR/deDHLNL ratio, the more mature the collagen cross-links. See also S1 Table in S1 File.

We compared the relative collagen content in the PC scars formed in the presence of the ACA or CA. The amount of collagen was calculated with respect to sulfated GAGs and with respect to the total protein content represented by the FTIR spectra’ amide II peak (S4 Fig in S1 File, Fig 6). Similar assays were done for the OCD scars formed in the ACA-treated and CA-treated rabbits (Fig 7). Table 6 summarizes one-way ANOVA tests to determine the statistical significance of observed differences between the analyzed groups.

**Table 6:** Analysis of differences between relative collagen contents and the maturity of collagen-cross-links in scar tissues formed in the PCs and osteochondral defects (OCD) in the CA-treated ACA-treated rabbits.

Scar tissue	Group	FTIR ratios		
		1064 cm^-1^ /1338 cm^-1^ GAGs/collagen	1549 cm^-1^/1338 cm^-1^ Amide II/collagen	1660 cm^-1^/1690 cm^-1^ PYR/de-DHLNL
**PC**	**8wk**	*F*(1, 9) = 0.02, *p* = 0.892	*F*(1, 9) = 0.159, *p* = 0.7	*F*(1, 9) = 0.003, *p* = 0.956
	**12wk**	*F*(1, 15) = 8.156, *p* = 0.012	*F*(1, 15) = 10.063, *p* = 0.006	*F*(1, 15) = 0.035, *p* = 0.854
**OCD**	**8wk**	*F*(1, 5) = 0.276, *p* = 0.621	*F*(1, 5) = 0.281, *p* = 0.619	*F*(1, 5) = 0.002, *p* = 0.965
	**12wk**	*F*(1, 22) = 1.043, *p* = 0.318	*F*(1, 22) = 0.407, *p* = 0.53	*F*(1, 22) = 4.331, *p* = 0.04

### FTIR assays of collagen cross-links in the PC and OCD scars

We also measured the maturity of collagen cross-links formed in the PC scar tissue in the presence of the ACA or CA. This was expressed as the FTIR-derived peaks’ ratio representing the mature PYR cross-link and immature de-DHLNL cross-link (Fig 6) [42]. Similar assays were done for the OCD scars formed in the ACA-treated and CA-treated rabbits (Fig 7). Table 6 summarizes one-way ANOVA tests to determine the statistical significance of observed differences between the analyzed groups.

### Assays of the flexion contracture

Since the ultimate purpose of the anti-fibrotic treatment with the ACA is to reduce scarring-associated stiffness in the injured joints, we measured the extent of flexion contracture in the ACA-treated and control rabbits. Specifically focusing on the 12wk group, we performed the superiority test (see Data analysis above) to analyze the ACA impact on the knee joint flexion contracture (Table 7, S11 Fig in S1 File). Our results indicate that the ACA effect is: 2.84/4.47 = 0.64, and the 95% CI is 0.41, 0.99, *p* = 0.047. Thus, compared to the control, the ACA-treatment reduced the uninjured/injured ratio (contraction), or the gap between the flexion contractures measured in the uninjured and the injured knees, and the reduction was significant (*p* = 0.047). In contrast, the 8wk group did not pass the superiority test. The ACA effect for this group was 9.82/8.6 = 1.14, and 95% CI 0.63, 2.06, *p* = 0.647 (Table 7, S12 Fig in S1 File).

**Table 7:** Summaries of tests to determine the ACA’s effects on the flexion contracture and parameters defining the quality of healing of the PTs (stress and Young’s modulus) and subchondral bone defects (Bv/Tv and BMD)^a^.

^a^Parameter	Duration	Group	GM	GM	GMR	The effect of ACA treatment (ACA/CA) with confidence levels and *p* values	
			Uninjured	Injured	Uni/Inj		
**Stress (MPa)**	**12wk**	CA	12.6	7.9	1.61	ACA/CA	0.98
		ACA	13.5	8.6	1.57	95% UCL	1.35
						*p =* 0.103	
	**8wk**	CA	15.7	3.9	4.03	ACA/CA	0.79
		ACA	13.5	4.2	3.20	95% UCL	1.21
						*p* = 0.040	
**Young’s modulus (MPa)**	**12wk**	CA	134.8	53.5	2.52	ACA/CA	1.02
		ACA	163.0	63.2	2.58	95% UCL	1.81
						*p* = 0.276	
	**8wk**	CA	258.8	32.6	7.94	ACA/CA	0.81
		ACA	233.7	36.4	6.41	95% UCL	1.69
						*p* = 0.161	
**Extension at 0.2 Nm (deg)**	**12wk**	CA	74.5	16.7	^b^4.47	ACA/CA	0.64
		ACA	75.7	26.6	^b^2.84	95% CI	(0.41, 0.99)
						*p* = 0.047	
	**8wk**	CA	65.0	7.6	^b^8.60	ACA/CA	1.14
		ACA	65.8	6.7	^b^9.82	95% CI	(0.63, 2.06)
						*p* = 0.647	
**Bv/Tv (%)**	**12wk**	CA	41.6	23.7	1.76	ACA/CA	0.97
		ACA	43.0	25.3	1.70	95% UCL	1.51
						*p* = 0.167	
	**8wk**	CA	51.4	15.0	3.43	ACA/CA	1.11
		ACA	50.2	13.2	3.80	95% UCL	2.01
						*p* = 0.366	
**BMD [g/cm** ^**3**^ **]**	**12wk**	CA	0.70	0.45	1.55	ACA/CA	1.38
		ACA	0.68	0.32	2.14	95% UCL	2.27
						*p* = 0.629	
	**8wk**	CA	1.08	0.47	2.30	ACA/CA	0.85
		ACA	0.87	0.44	1.95	95% UCL	1.66
						*p* = 0.168	

^a^ Presented parameters were measured in uninjured and injured legs of rabbits from the CA-treated and ACA-treated groups (GMs are presented). Within each group, potential changes of these parameters in the injured legs were expressed as the ratios of values calculated for the uninjured and the injured legs (GMR). Subsequently, the effect of the ACA on these ratios was evaluated (ACA/CA) and presented with p values.

^b^ Joint contracture values defined as the ratio of knee extension of uninjured joint and that of contralateral injured joint. Please note that the starting point to measure knee extension was positioning the femur and the tibia at right angle (S5 Fig in S1 File). At this starting position, the value of extension was set to 0°. Subsequently, the torque was applied to the value of 0.2Nm and the extension angle (deg) was recorded. Non-stiff knees (uninjured) reached relatively high extension values while stiff (injured) knees reached relatively low extension values. Hence, large uninjured/injured ratios of measured extensions indicate a relatively large contracture while small uninjured/injured ratios of measured extensions indicate a relatively small contracture.

### Impact of the ACA on the quality of healing of the PTs

We also measured the PT mechanical properties to determine the quality of their healing in the presence or absence of the ACA. Focusing on the PT ultimate stresses from the 12wk group as an example, we found, in brief, that the GMs for the ACA and CA groups’ uninjured PTs were ~13 MPa, indicating proper randomization (Table 7, S11 Fig in S1 File). In contrast, the GMs for injured PTs in both groups had lower values. The GM dropped from 12.6 to 7.9 in the CA group and from 13.5 to 8.6 in the ACA group. The corresponding GMRs were 12.6/7.9 = 1.61 (CA) and 13.5/8.6 = 1.57 (ACA) (Table 7, S11 Fig in S1 File).

Considering the ratios for the CA group (1.61) and ACA group (1.57), in the CA group, the GM of the uninjured PT was 61% higher, on average, than the GM of the injured one; and in the ACA group, the GM of the injured PT was 57% higher, on average, than the GM of the injured one. Thus, it seems that the ACA treatment reduced the "gap" between uninjured and injured tendons from 1.61 to 1.57. The formal treatment effect is 1.57/1.61 = 0.98 (the smaller the value, the more significant the effect).

According to the hypothesis we presented in our original pre-experimental plan (see Data analysis), we are willing to accept up to 1.25 (25% increase of the "gap"). To formally test this non-inferiority hypothesis, we put a 95% one-sided (upper) confidence limit to the 0.98 value, resulting in a formal treatment effect of 1.35. So, even though our best estimate is that the ACA slightly reduces the uninjured-to-injured difference, we cannot exclude the possibility that it increases it up to 35%. Therefore, we have not proven non-inferiority (the associated *p*-value is 0.103, Table 7 and S11 Fig in S1 File). We reached a similar conclusion for the results of measurements of Young’s modulus of the PTs from the 12wk group (Table 7, S11 Fig in S1 File).

For the 8wk group (Table 7, S12 Fig in S1 File), we have proven non-inferiority for the ultimate stress parameter (*p* = 0.04). The Young’s modulus, however, did not pass the non-inferiority test (*p* = 0.161).

### Biomineralization in the presence of the ACA

We also performed the non-inferiority test to compare the mineralization of subchondral bone in the ACA group versus the CA group. Assays of the Bv/Tv and the BMD parameters in the OCDs showed a drop in mineralization in both the ACA and CA groups (Table 7, S13 and S14 Figs in S1 File). The Bv/Tv and BMD values for the ACA and CA groups’ uninjured bones were similar, indicating good randomization. The ratios of parameters measured in the uninjured and injured sites were reasonably similar (Table 7, S13 and S14 Figs in S1 File). Despite the close similarities, both Bv/Tv and BMD measured for the ACA group did not pass the non-inferiority tests (Table 7).

### Impact of the ACA on internal tissues and organs

Because of the dynamic exchange of macromolecules between the intraarticular space and the lymphatic and circulatory systems, we analyzed whether continuous administration of the ACA had any negative impact on distant tissues and organs. Histology of crucial tissues and organs collected from all rabbits from the 8wk and the 12wk groups did not show any structural abnormalities or excess inflammatory cells. Supplementary figures present histological images of assayed specimens from the 8wk group treated with the ACA or CA highest concentration (S15–S22 Figs in S1 File).

## Discussion

Surgical and accidental injuries to articular joints may lead to arthrofibrosis. Because of the significant burden of arthrofibrosis, therapeutic approaches are needed to effectively and safely reduce excessive posttraumatic scarring [56, 57]. Our earlier research demonstrated that targeting collagen fibrillogenesis with a novel ACA reduces scar formation and decreases joint stiffness [16]. While we have previously demonstrated the primary mechanism of the ACA-dependent inhibition of collagen fibril formation, the overall effects of applying this antibody have not been studied thus far. Hence, studies presented here aim to define the crucial effects of blocking collagen fibrillogenesis in the posttraumatic model of arthrofibrosis.

### Impact of the ACA on collagen turnover

Our results on serum concentration of HP measured across various stages of the healing process indicate that it increased significantly between time-0 and week-4 in the ACA-treated group. We associate this increase, in part, with the ACA-mediated upsurge of free collagen molecules that have not been incorporated into collagen fibrils.

While assays of serum HP provide information on the degradation of collagenous proteins, concentration of serum CP reflects the biosynthesis of procollagen I [58]. In contrast to the control, we determined that the CP serum concentration in the ACA group changed significantly across selected time points. This observation was somewhat unexpected because the ACA does not affect the production of procollagen I. However, it is essential to point out that in normal conditions procollagen I molecules, with their C-terminal propeptides, associate transiently with matrix-bound collagen microfibrils. Studies have demonstrated that CPs present on the surfaces of such fibrils regulate their shape [59]. Thus, it is conceivable that the lack of significant changes in the serum concentration of the CPs across measured time points in control group was due to the trapping of the procollagen I molecules via their binding with tissue microfibrils. Hence, we hypothesize that the ACA reduces the amount of microfibril-bound procollagen I, thereby accelerating its degradation and increasing the CP’s serum concentration. Our recent study result showing that the ACA may bind its epitope in the microfibrils, in addition to binding free collagen molecules, supports this hypothesis [11, 60].

In the ACA group, the CP’s serum concentration peak and the HP’s concentration peak coincided. These data suggest that the first four post-surgery weeks represent a dynamic phase of collagen biosynthesis and degradation in the model of arthrofibrosis utilized here. These data are consistent with collagen metabolism kinetics measured after injuries to the bone, tendon, and skin [61–63].

Unlike HP and CP, cross-linked collagen peptides present in the sera are derived solely from collagen fibrils. Thus, assays of collagen I-derived XLs provided an opportunity to measure the dynamics of matrix remodeling in the collagen I-rich joint tissues in the presence of the ACA. We demonstrated that the increase of the XL serum concentration was significantly higher in the ACA-treated group than in control. We predict that this rapid increase in the ACA-treated rabbits reflects a robust remodeling phase of the wound healing process. Due to the ACA-dependent reduction of the new collagen fibrils that stabilize the scar tissue, the remodeling phase likely had an earlier onset. In support of this postulation, a study of skin wound healing demonstrated that new collagen fibrils appeared in the wounded site three days post-injury [64]. Hence, we suggest that the ACA-dependent acceleration of tissue remodeling may represent an additional mechanism by which this antibody reduces the scar formation and improves joint mobility after traumatic injuries.

### The ACA-dependent reduction of scar collagen

Histological and FTIR assays of collagen matrices in the scar tissues formed in the PCs and within the OCDs support the ACA-dependent reduction of collagen fibrils. Histological assays of the PCs from the control group’s injured joints demonstrated a significant increase in the thin fibrils formed in the injury sites [16, 21]. In contrast, we observed no significant increase in the thin fibrils in the injured PCs treated with the ACA, most likely due to the ACA-dependent attenuation of collagen fibrillogenesis [16].

Unlike in the PCs, in the pannus-like tissue formed around and within the OCDs, we observed no differences in the relative contents of corresponding collagen subpopulations, with different birefringence, in the ACA and control groups. To explain this dissimilarity, we point out that while in the PCs the scar tissue is mainly formed by local fibroblasts that produce collagen I, in the OCD, the pannus-like scar tissue is formed *de novo* with potential involvement of chondrocyte-like cells [16, 21, 65]. As such, this scar includes collagen II fibrils, in addition to collagen I [66, 67]. Since collagen II fibrils are relatively thin, they demonstrate green birefringence when stained with picrosirius red and observed under polarized light [68]. Because the ACA does not interact with collagen II, it does not interfere with collagen II fibrillogenesis. Therefore, we postulate that the ACA impact on pannus-like structure formation is less prominent than on strictly collagen I-based matrices.

### ACA-associated changes in collagen cross-links

We also studied the ACA effects on collagen fibrils cross-links. The FTIR-based studies showed no effect of the ACA on the mature PYR/immature de-DHLNL cross-link ratio in injured PCs. In contrast, we observed a statistically significant increase in this ratio in the fibrotic tissue formed in the pannus-like tissue formed within the OCDs of the ACA-treated 12wk group.

The increase of this ratio could reflect the decrease of the denominator, i.e., the immature cross-links, due to the ACA-dependent reduction of new collagen I-based fibrils that contain de-DHLNL cross-links [69]. However, we cannot exclude a possibility that the numerator increase, i.e., the PYR cross-links, could have raised the PYR/de-DHLNL ratio. Since we observed this rise in the 12wk group but not in the 8wk group, we postulate that mechanical stimulation enabled by joint remobilization could have partially increased the PYR cross-links content [70]. Considering the dynamic nature of changes in developing scar tissues, the observed increase in the PYR/de-DHLNL ratio represented, most likely, the net changes in their numerator and denominator components.

### Changes in joint stiffness

Since the excessive formation of collagen-rich scar tissue stiffens the knee joint structures, most notably the PC, we analyzed whether the ACA-mediated changes in matrix remodeling and the collagen content reduce joint stiffness. Utilizing the superiority test, we confirmed our earlier observations and demonstrated that the ACA reduces the flexion contracture significantly in the 12wk group [16].

We did not see significant differences between the ACA-treated and the CA-treated rabbits from the 8wk group analyzed immediately after removing the K-wires. As indicated earlier, the immobilization itself stiffens the joints by mechanisms other than scarring, and a recovery period is needed to reduce this impact [24–26, 71]. Utilizing the same animal model, Hildebrand *et al*. showed that even without any intervention, the flexion contracture decreases gradually following the K-wire removal and plateaus after about 16 weeks without reaching a typical value of an uninjured knee [71]. Consequently, significantly reduced stiffness in the 12wk group treated with the ACA, but not with CA, suggests that this antibody accelerates the recovery process compared to the CA-treated control. Considering faster remodeling and decreased amounts of collagen material in the PCs, we propose that the ACA-mediated inhibition of collagen fibrillogenesis is a linchpin of the accelerated recovery.

### The quality of healing in the presence of ACA

Although reduced flexion contracture in the ACA group is a positive therapeutic outcome, the quality of joint tissue healing needed to be assessed since the ability of ACA to reduce fibrillogenesis could negatively impact the quality of healed tissues.

We hypothesized that the ACA treatment is not substantially worse than a control treatment. Since the joint tissue mechanical properties define their quality, we measured the ultimate stress and Young’s moduli of healed PTs. We also measured the mineralization of the OCDs as a parameter defining the healing of the bone defects. Our results indicate that the mechanical and mineralization parameters were similar in the ACA and control groups, albeit we did not prove non-inferiority of the ACA treatment. Future experiments with larger animal groups will clarify the non-inferiority issue.

### Effects of the ACA administration on distant organs

A crucial requirement of any antibody-based therapy is a lack of significant off-target cross-reactivity and any significant negative impact on vital tissues and organs. Our earlier studies demonstrated that the ACA does not cross-react with uninjured collagen-rich tissues undergoing no active fibrillogenesis [14]. Here, the dose escalation studies demonstrated that a relatively high concentration of the ACA does not cause any unwanted changes in crucial tissues and organs, despite long-term treatment. In particular, the lack of the ACA effects on collagen I-rich tissues, including tendon, sciatic nerve, muscle, lung, and others, indicates that this antibody has target-specific properties. Normal cellular and biochemical parameters of the blood of the ACA-treated rabbits further indicate the safety of the ACA treatment.

Our study presented here is the first report that defines crucial mechanisms for reducing posttraumatic arthrofibrosis by directly targeting collagen fibrillogenesis. Detailed assays of the collagen metabolism and structure of collagen-rich matrices demonstrate the high specificity and safety of the ACA. Consequently, this study paves the way to move the anti-fibrotic technology presented here toward clinical application.

### Limitations of the study

This study has a few limitations that should be considered when interpreting the results. First, the arthrofibrosis model we employed here does not represent all injury types caused by trauma to the joints. For instance, we did not introduce bone fractures, which frequently occurs due to joint injury. As healing of bone fractures may impact the healing process of joint tissues, future models should include them to study the utility of anti-fibrotic agents. Second, we realize that although the number of animals in the 8wk groups was appropriate for the dose escalation studies, the number was not adequate for mechanical and biochemical assays of scar tissues. Consequently, results of these assays should be interpreted considering this limitation. Third, although a pump-based system provided a reliable and controllable ACA-delivery platform, we realize its limits for clinical use. Hence, in the future, clinically-relevant biomaterials should be evaluated for sustained delivery of the ACA.

## Supporting information

S1 File (PDF)
