# Supplementary material for: Mechanisms of reducing joint stiffness by blocking collagen fibrillogenesis in a rabbit model of posttraumatic arthrofibrosis
Source: PLoS One. 2021 Sep 7;16(9):e0257147. doi: 10.1371/journal.pone.0257147 (PMC8423260; doi:10.1371/journal.pone.0257147)
Supplement: S1 File — (PDF) [file pone.0257147.s001.pdf]

Supplementary Materials

Mechanisms of reducing joint stiffness by blocking collagen fibrillogenesis in a rabbit model of posttraumatic arthrofibrosis.

Andrzej Steplewski [1], Jolanta Fertala [1], Ryan E. Tomlinson [1], Mark L. Wang [1,2], Allison Donahue [3], William Vincent Arnold [1,2], Michael Rivlin [1,2], Pedro K. Beredjiklian [1,2], Joseph A. Abboud [1,2], Surena Namdari [1,2], and Andrzej Fertala [1*]

[1]Department of Orthopaedic Surgery, Sidney Kimmel Medical College, Thomas Jefferson University, Philadelphia, PA
[2]Rothman Institute of Orthopaedics, Thomas Jefferson University Hospital, Philadelphia, PA
[3]College of Medicine, Drexel University, Philadelphia, PA

***Corresponding author**
e-mail: andrzej.fertala@jefferson.edu.

Supplementary Table S1. Fourier-transform infrared (FTIR) spectroscopy parameters applied in collagen assays.

**Target**	**Band position [cm^{-1}]**	**Bond vibrations**	**Purpose**
Sulfated GAGs	1064	SO^{-3} vibration	To provide a reference for collagen changes
Protein-specific amide II peak	1549	N-H bending and the C-N stretching vibration	To provide a total protein reference for collagen changes
Collagen	1338	CH_2 wagging vibration of proline side chains	To measure relative changes in collagen content
PYR cross-link	1660	A constituent of amide I peak	To measure mature pyridinoline cross-links
de-DHLNL cross-link	1690	A constituent of amide I peak	To measure immature dehydro-dihydroxynorleucine cross-links, an immature PYR precursor.

Supplementary Figures:

Supplementary Figure S1. Crucial surgical steps to generate knee injury in a rabbit model of arthrofibrosis. A, Getting an access to the knee cavity. B, Drilling a hole in the tibia (Tb) to install a Kirschner wire (Kw). C, Placing a loop of the Kw over a femur (Fe), D, Fixing the Kw with a nut placed over the tibia. E, Installing a pump (P) connected to the knee cavity via a silicone tube (St). F, An X-ray image of the operated leg immobilized in the flexed position with a Kw.

Supplementary Figure S2. A schematic of the experimental design of the study.

Supplementary Figure S3. A schematic depicting the premises of assays of collagen metabolites. A, A procollagen I molecule with intact N propeptides (NP) and C propeptides (CP); hydroxyproline residues present in the triple-helical domain are also indicated (HP). B, A collagen I molecule generated by extracellular cleavage of the NP and the CP; here, we will analyze the CP of procollagen I. C, A typical staggered arrangement of collagen molecules incorporated into a fibril. Collagen cross-links (XL) that link these collagen molecules are indicated with X. D, Collagen fragments formed due to turnover/degradation of collagen fibrils. Note that all collagen triple helix-derived fragments will include HP, and some will retain the collagen cross-links.

Supplementary Figure S4. FTIR spectroscopy of the pannus-like scar tissue formed within OCDs. A, A microscopic image of a selected ROI. A dotted line marks the border between the bone (B) and fibrotic pannus-like tissue (FT). B, A corresponding map of the average intensity of the FTIR signals. C, A representative FTIR spectrum (black line) with fitted peaks (red line). The peaks utilized in this study are indicated.

Supplementary Figure S5. A custom-made device to measure the flexion contracture of the knee joints. Clamping of the femur (Fe) and tibia (Tb) is indicated. The arrow shows the movement of the femur.

Supplementary Figure S6. A. Isolation of the patellar tendon (PT)-tibia (Tb) complex. B. A setup of the complex seen in A for the mechanical tests. The insert depicts the PT in which the arrow indicates the healed defect created during the initial surgery.

Supplementary Figure S7. Cell parameters of the blood samples collected at indicated time points from the 8wk ACA-treated group (red lines) and control (black lines).

Supplementary Figure S8. Biochemical parameters of the blood samples collected at selected time points from the 8 wk ACA-treated group (red lines) and control (black lines).

Supplementary Figure S9. Cell parameters of the blood samples collected at various time points from rabbits maintained for 12 weeks after surgery in the presence (red lines) or the absence (black lines) of the ACA.

Supplementary Figure S10. Biochemical parameters of the blood samples collected at various time points from rabbits maintained for 12 weeks after surgery in the presence (red lines) or the absence (black lines) of the ACA.

Supplementary Figure S11. A graphic representation of differences in mechanical parameters of the knee joints and PTs in the ACA-treated (□ and ■) and the CA-treated (○ and ●) rabbits from the 12wk group. The left column shows results of measurements of uninjured (Un) and injured (In) legs. The GMs and 95% confidence intervals are indicated. The left panels show the Un/In ratios of analyzed parameters.

Supplementary Figure S12. A graphic representation of differences in mechanical parameters of the knee joints and PTs in the ACA-treated (□ and ■) and the CA-treated (○ and ●) rabbits from the 8wk group. The left column shows results of measurements of uninjured (Un) and injured (In) legs. The GMs and 95% confidence intervals are indicated. The left panels show the Un/In ratios of analyzed parameters.

Supplementary Figure S13. A graphic representation of differences in mineralization of osteochondral defects in the ACA-treated (□ and ■) and the CA-treated (○ and ●) rabbits from the 8wk group. The left column shows results of measurements of uninjured (Un) and injured (In) legs. The GMs and 95% confidence intervals are indicated. The left panels show the Un/In ratios of analyzed parameters.

Supplementary Figure S14. A graphic representation of differences in mineralization of osteochondral defects in the ACA-treated (□ and ■) and the CA-treated (○ and ●) rabbits from the 12wk group. The left column shows results of measurements of uninjured (Un) and injured (In) legs. The GMs and 95% confidence intervals are indicated. The left panels show the Un/In ratios of analyzed parameters.

Supplementary Figure S15. Histology of brain tissues. Symbols: ScWm; subcortical white matter, Odg; oligodendroglia, Pc; Purkinje cells.

Supplementary Figure S16. Histology of stomach and esophagus. Symbols: Mm; mucous membrane, Sm; submucosa, Lp; lamia propia, Sse-k; stratified squamous epithelium-keratinized.

Supplementary Figure S17. Histology of aorta and heart muscle. Symbols: Tum; tunica media, Myc; myocardium.

Supplementary Figure S18. Histology of the lung tissue and intestines. Symbols: Ig; intestinal glands. Because the lungs were not inflated prior to the fixation, the lung tissue appears compact.

Supplementary Figure S19. Histology of liver and kidney. Symbols: Hl; hepatic lobule, Rc; renal cortex.

Supplementary Figure S20. Histology of spleen and thymus. Symbols: Cr-Tc; cortex thymic cells, Ln; lymphoid nodule.

Supplementary Figure S21. Histology of the sciatic nerve and the Achilles tendon. Symbols: Nf; neural filaments, Cf; collagen fibers.

Supplementary Figure S22. Histology of testis and ovaries. Symbols: CST; convoluted seminiferous tubules, Grn; granulosa cells, Ti; theca interna, Oc; oocyte.

SUPPLEMNTARY FIGURES

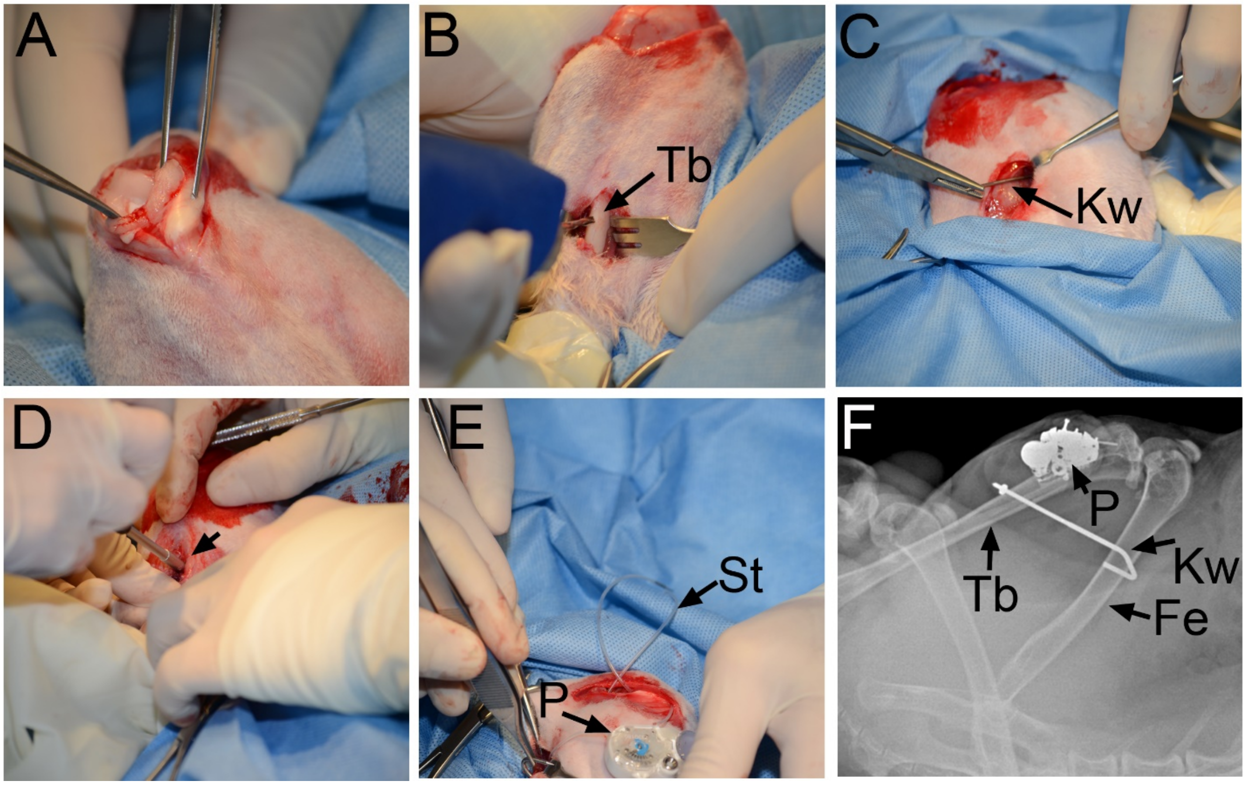

Figure S1

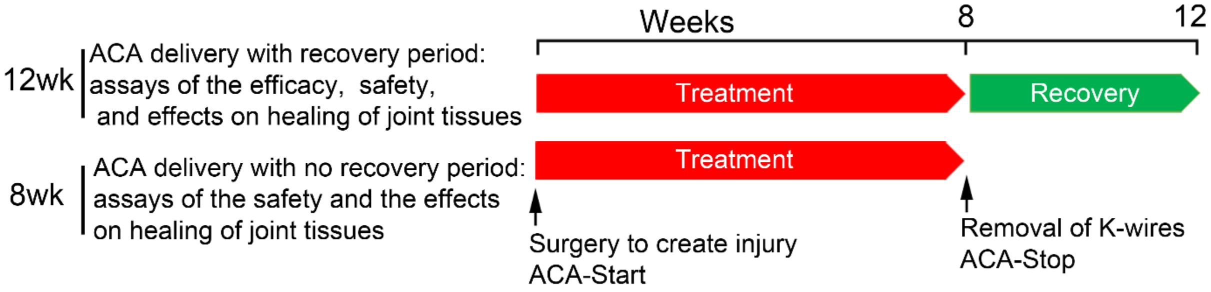

Figure S2

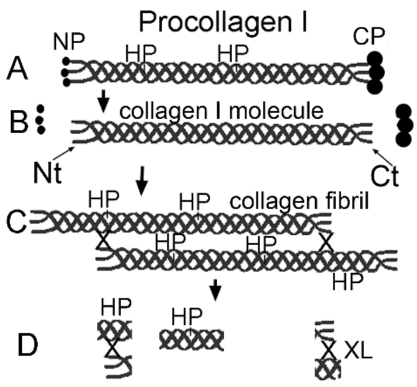

Figure S3

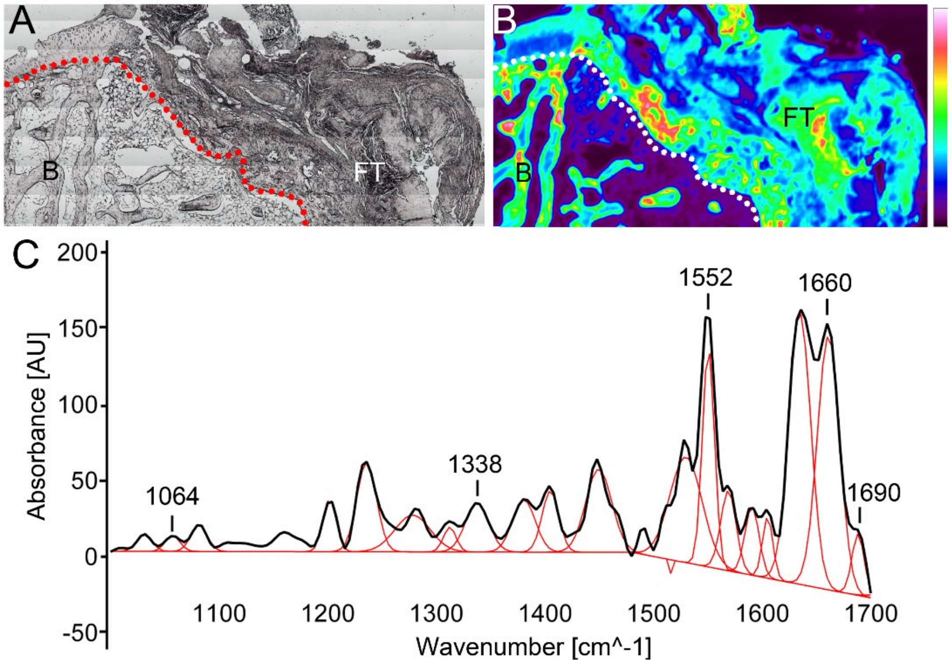

Figure S4

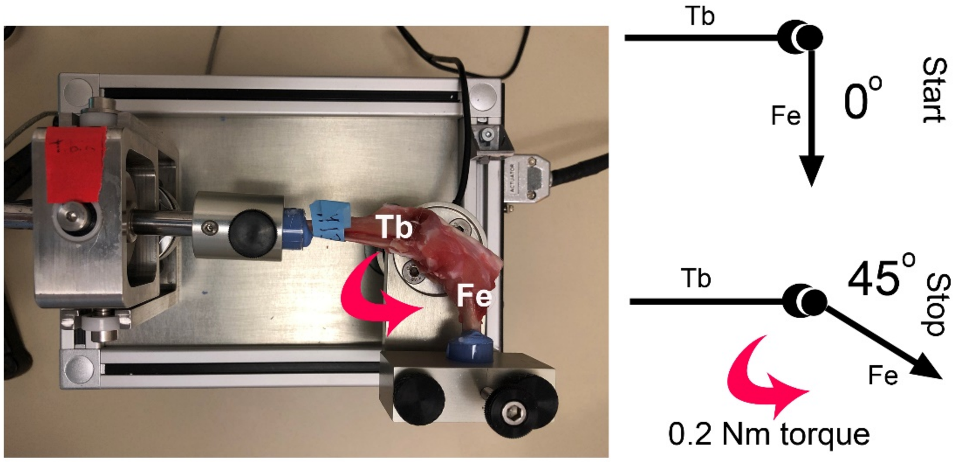

Figure S5

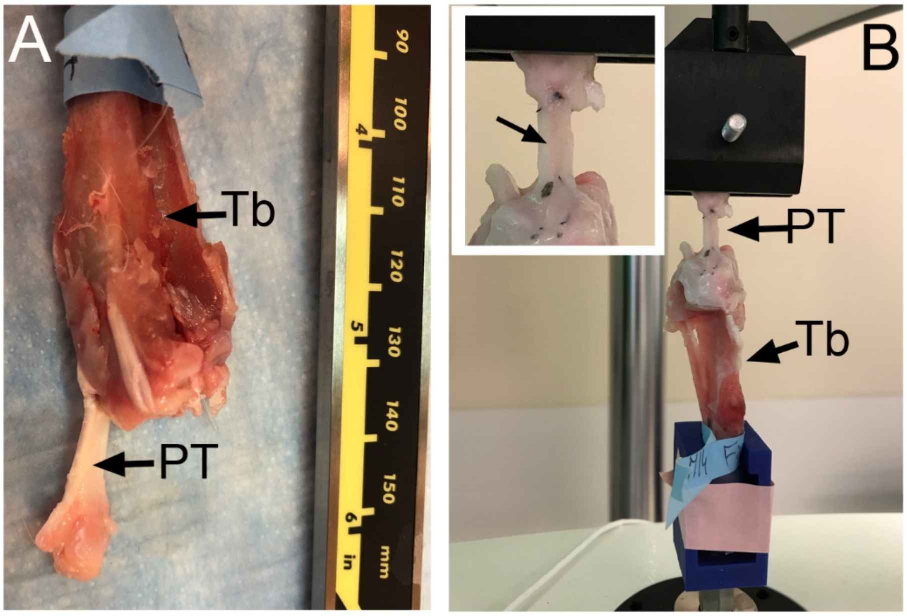

Figure S6

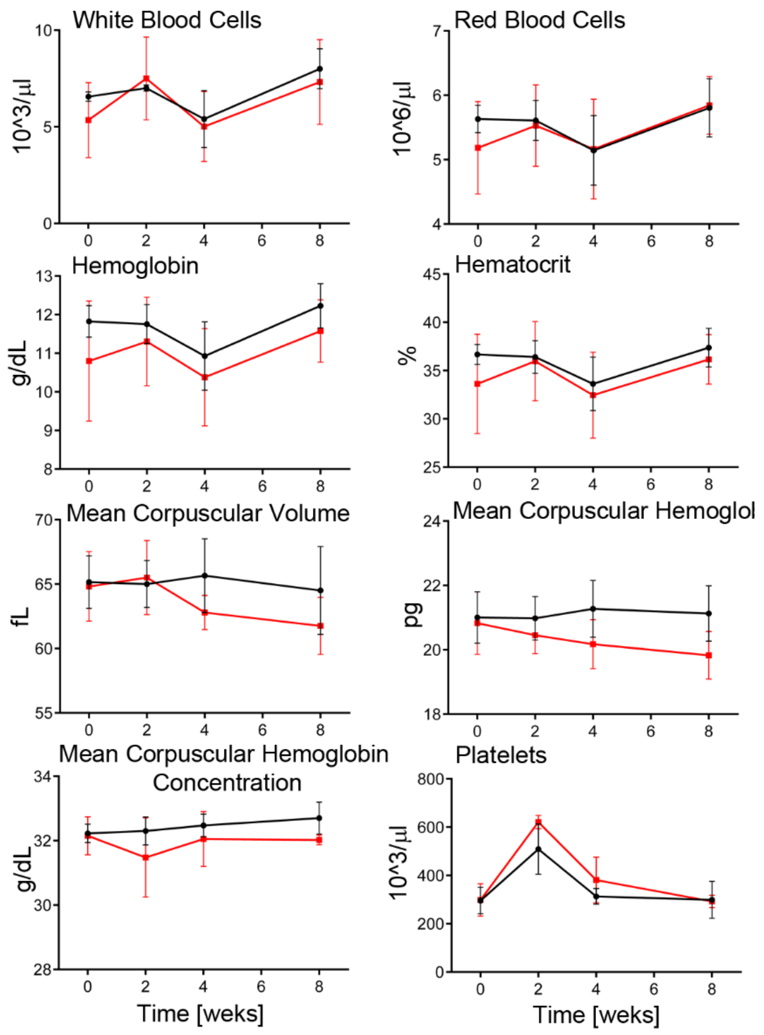

White Blood Cells
10^3/µl
Red Blood Cells
10^6/µl
Hemoglobin
g/dL
Hematocrit
%
Mean Corpuscular Volume
fL
Mean Corpuscular Hemoglol
pg
Mean Corpuscular Hemoglobin Concentration
g/dL
Platelets
10^3/µl
Time [weks]
Time [weks]

Figure S7

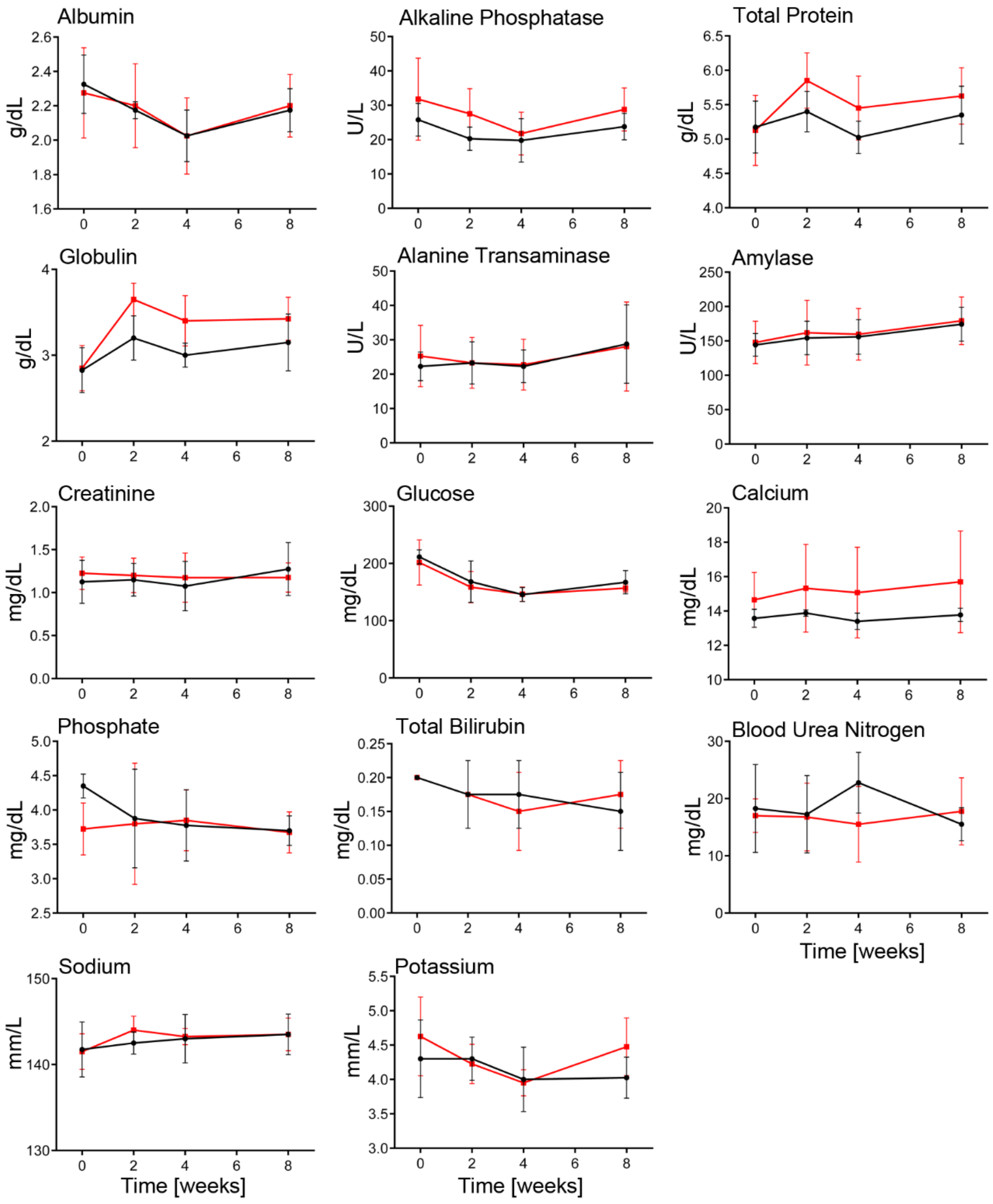
Albumin
g/dL
Alkaline Phosphatase
U/L
Total Protein
g/dL
Globulin
g/dL
Alanine Transaminase
U/L
Amylase
U/L
Creatinine
mg/dL
Glucose
mg/dL
Calcium
mg/dL
Phosphate
mg/dL
Total Bilirubin
mg/dL
Blood Urea Nitrogen
mg/dL
Time [weeks]
Sodium
mm/L
Potassium
mm/L
Time [weeks]
Time [weeks]

Figure S8

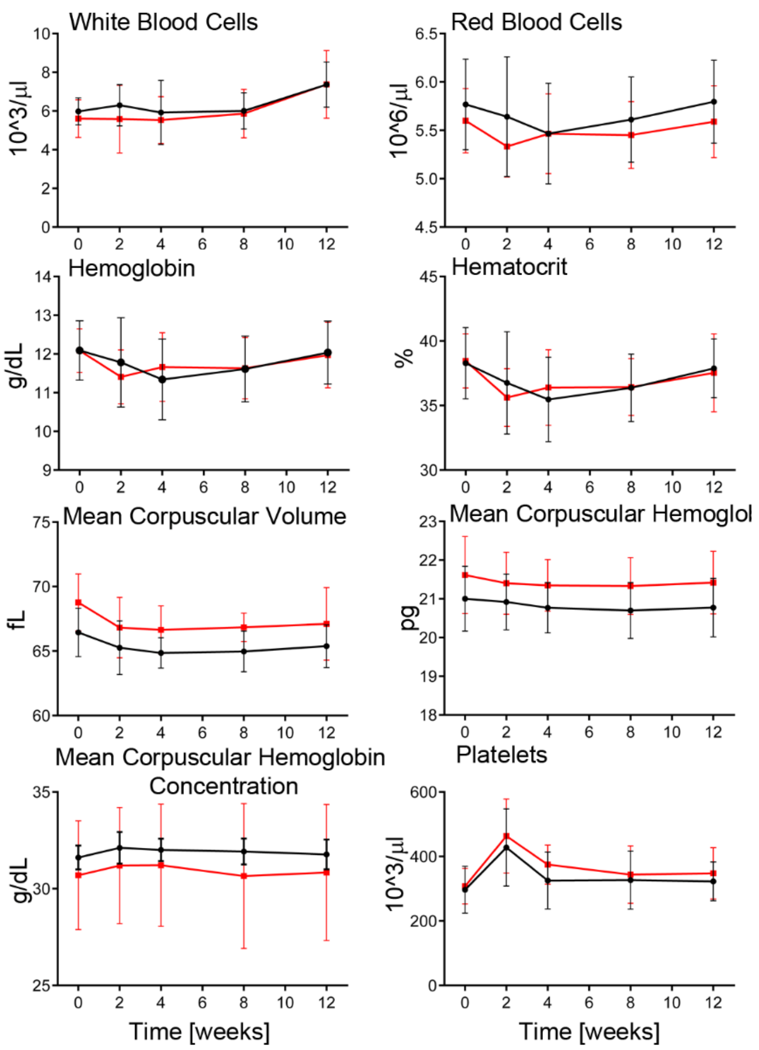

White Blood Cells
10^3/µl
Red Blood Cells
10^6/µl
Hemoglobin
g/dL
Hematocrit
%
Mean Corpuscular Volume
fL
Mean Corpuscular Hemoglol
pg
Mean Corpuscular Hemoglobin Concentration
g/dL
Platelets
10^3/µl
Time [weeks]
Time [weeks]

Figure S9

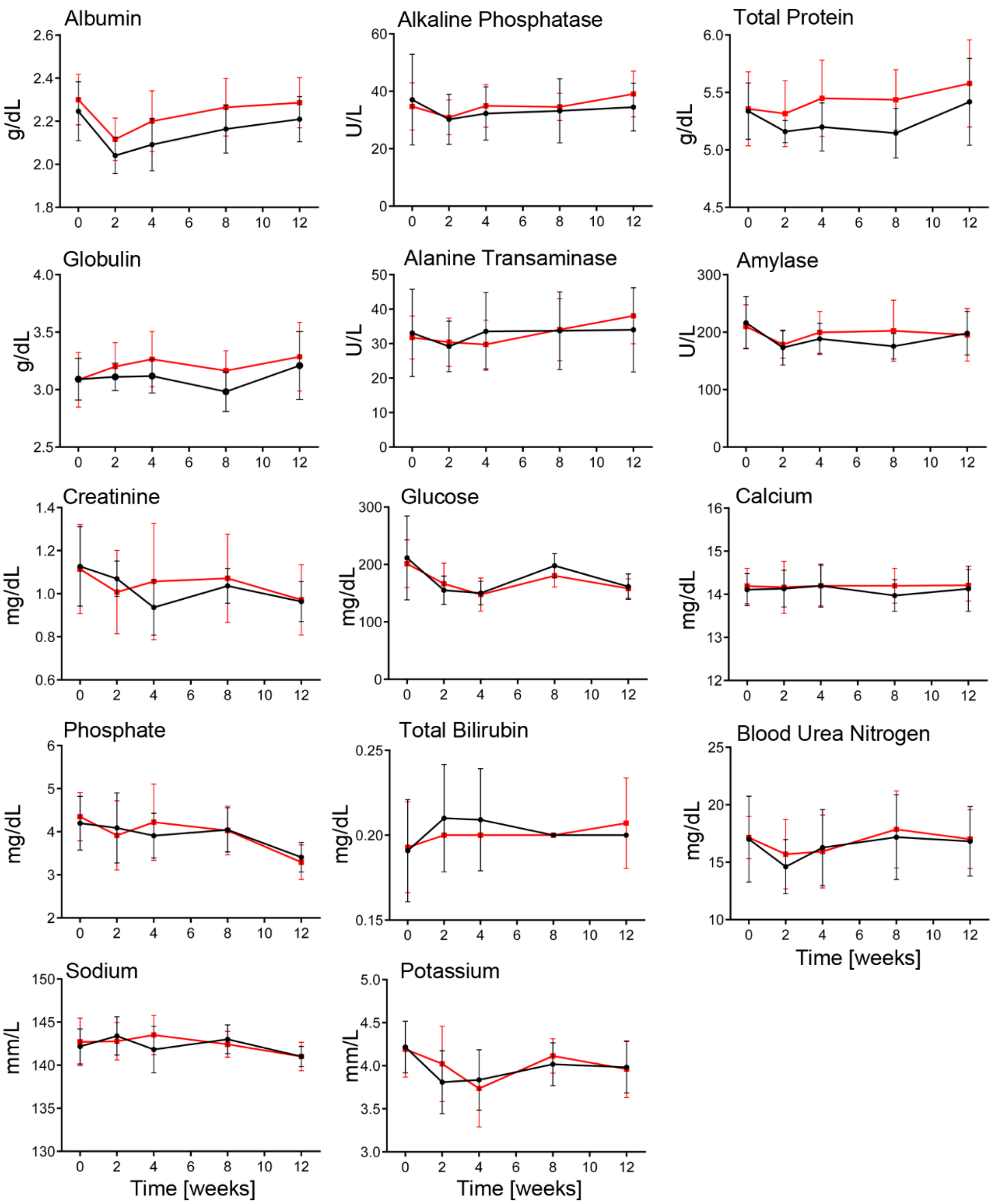
Albumin
g/dL
Alkaline Phosphatase
U/L
Total Protein
g/dL
Globulin
g/dL
Alanine Transaminase
U/L
Amylase
U/L
Creatinine
mg/dL
Glucose
mg/dL
Calcium
mg/dL
Phosphate
mg/dL
Total Bilirubin
mg/dL
Blood Urea Nitrogen
mg/dL
Time [weeks]
Sodium
mm/L
Potassium
mm/L
Time [weeks]
Time [weeks]

Figure S10

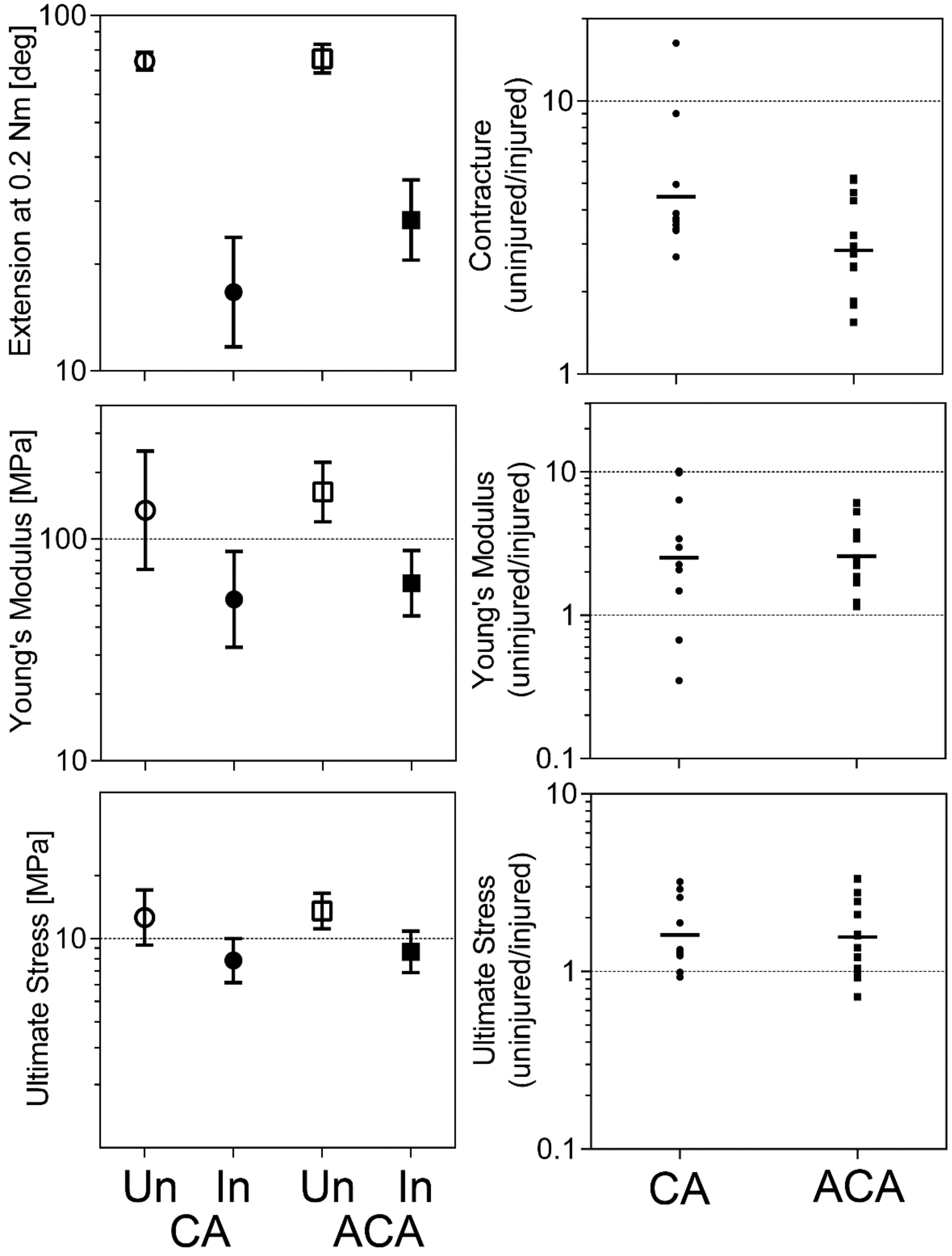

100
Extension at 0.2 Nm [deg]
10
Contracture
(uninjured/injured)
10
1
Young's Modulus [MPa]
100
10
Young's Modulus
(uninjured/injured)
10
1
0.1
Ultimate Stress [MPa]
10
Ultimate Stress
(uninjured/injured)
10
1
0.1
Un In Un In
CA ACA
CA ACA

Figure S11

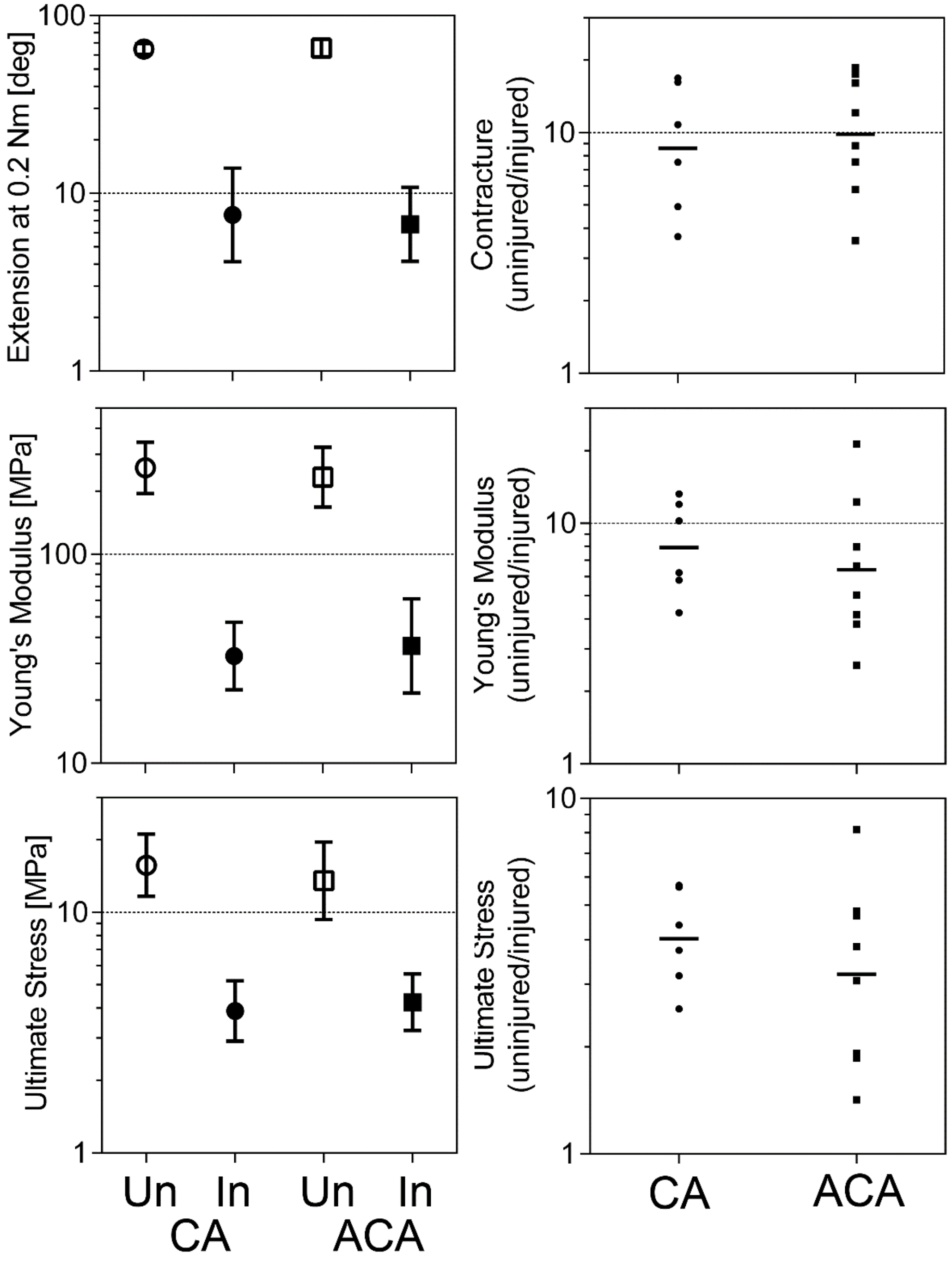

Extension at 0.2 Nm [deg]
100
10
1
Contracture
(uninjured/injured)
10
1
Young's Modulus [MPa]
100
10
Young's Modulus
(uninjured/injured)
10
1
Ultimate Stress [MPa]
10
1
Ultimate Stress
(uninjured/injured)
10
1
Un
In
Un
In
CA
ACA
CA
ACA

Figure S12

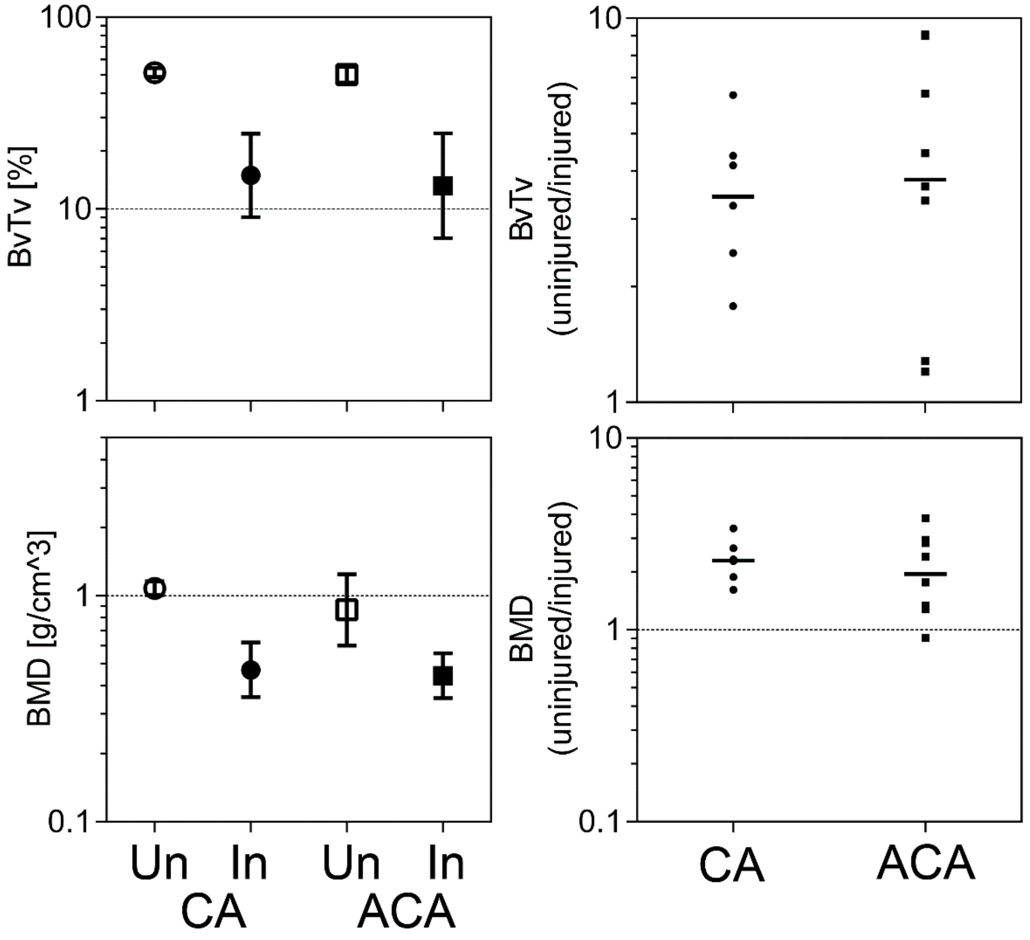
100
10
1
BvTv [%]
10
1
BvTv
(uninjured/injured)
1
0.1
BMD [g/cm^3]
10
1
0.1
BMD
(uninjured/injured)
Un
In
Un
In
CA
ACA
CA
ACA

Figure S13

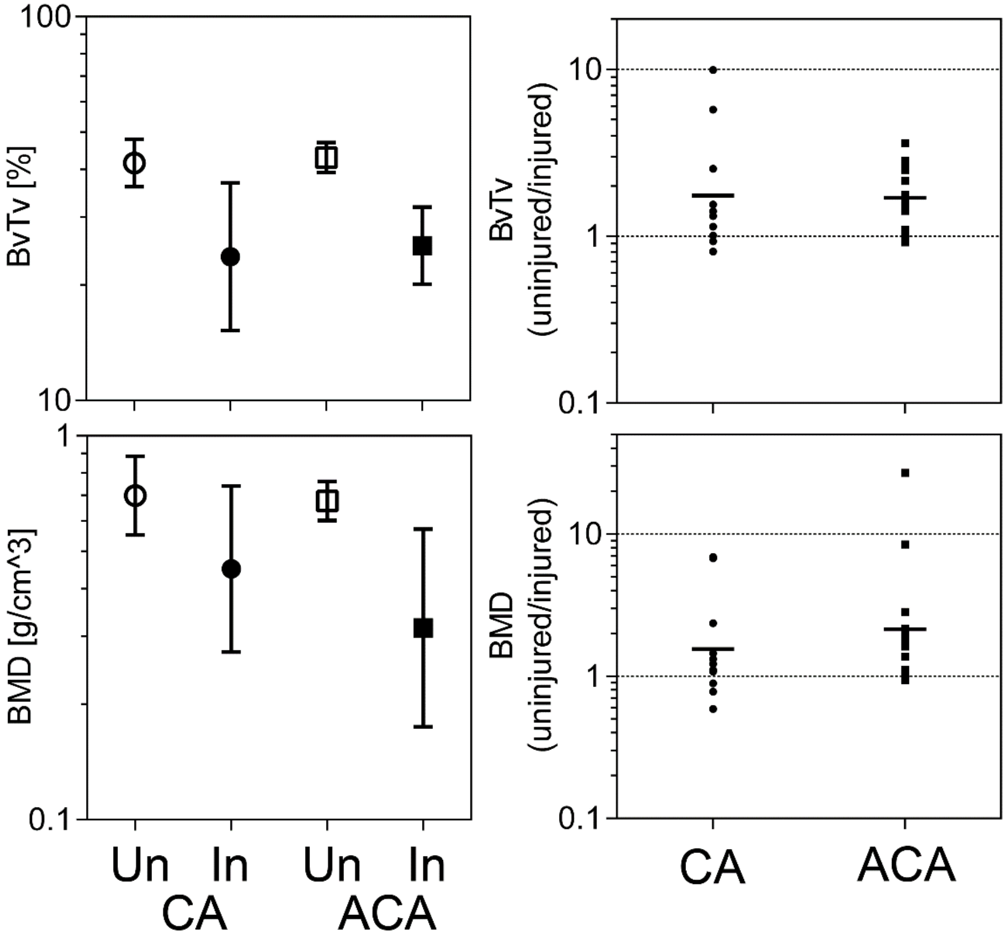
100
10
BvTv [%]
1
0.1
BMD [g/cm^3]
Un
In
Un
In
CA
ACA
10
1
0.1
BvTv
(uninjured/injured)
BMD
(uninjured/injured)
CA
ACA

Figure S14

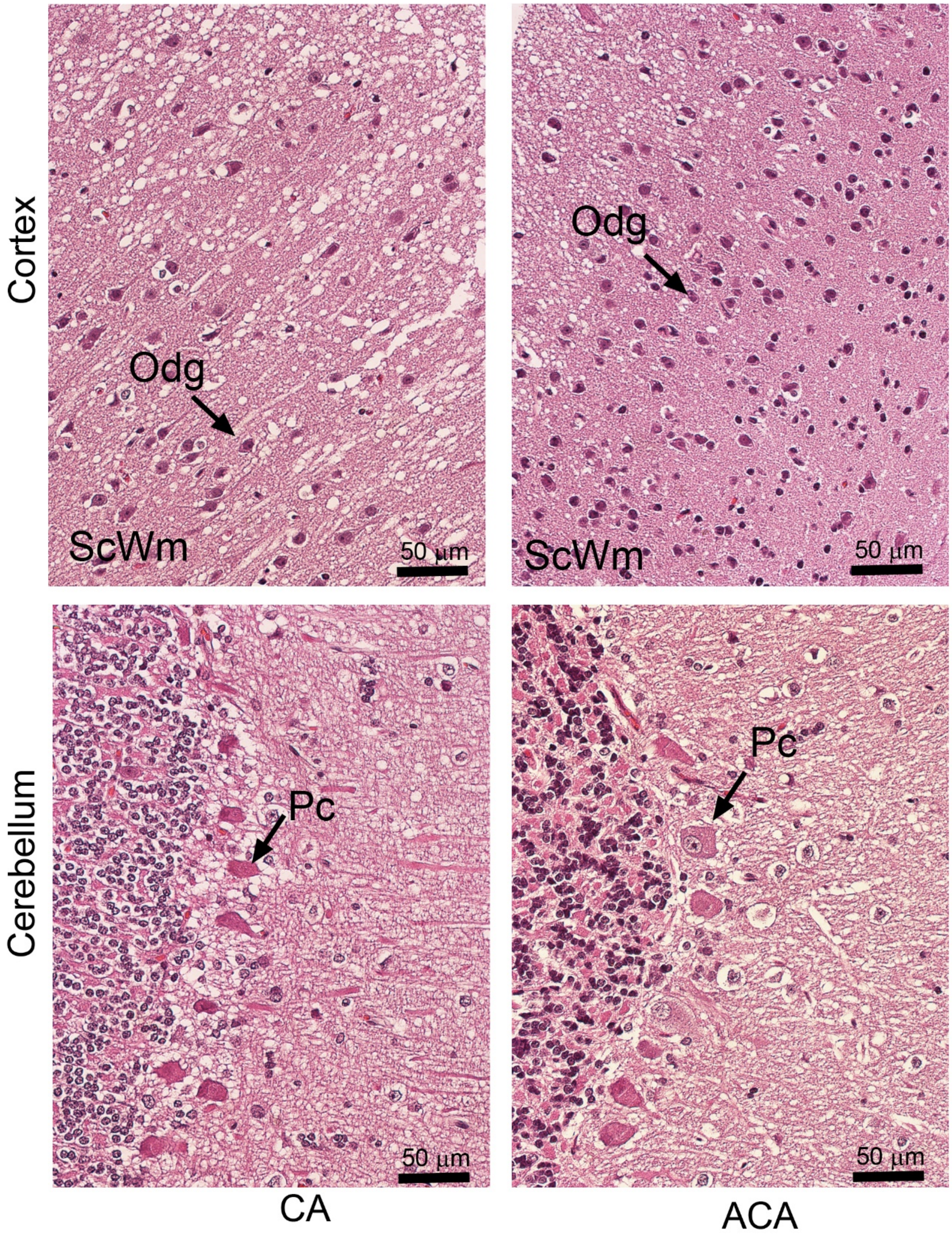
Cortex
Odg
ScWm
50 µm
Odg
ScWm
50 µm
Cerebellum
Pc
50 µm
Pc
50 µm
CA
ACA

Figure S15

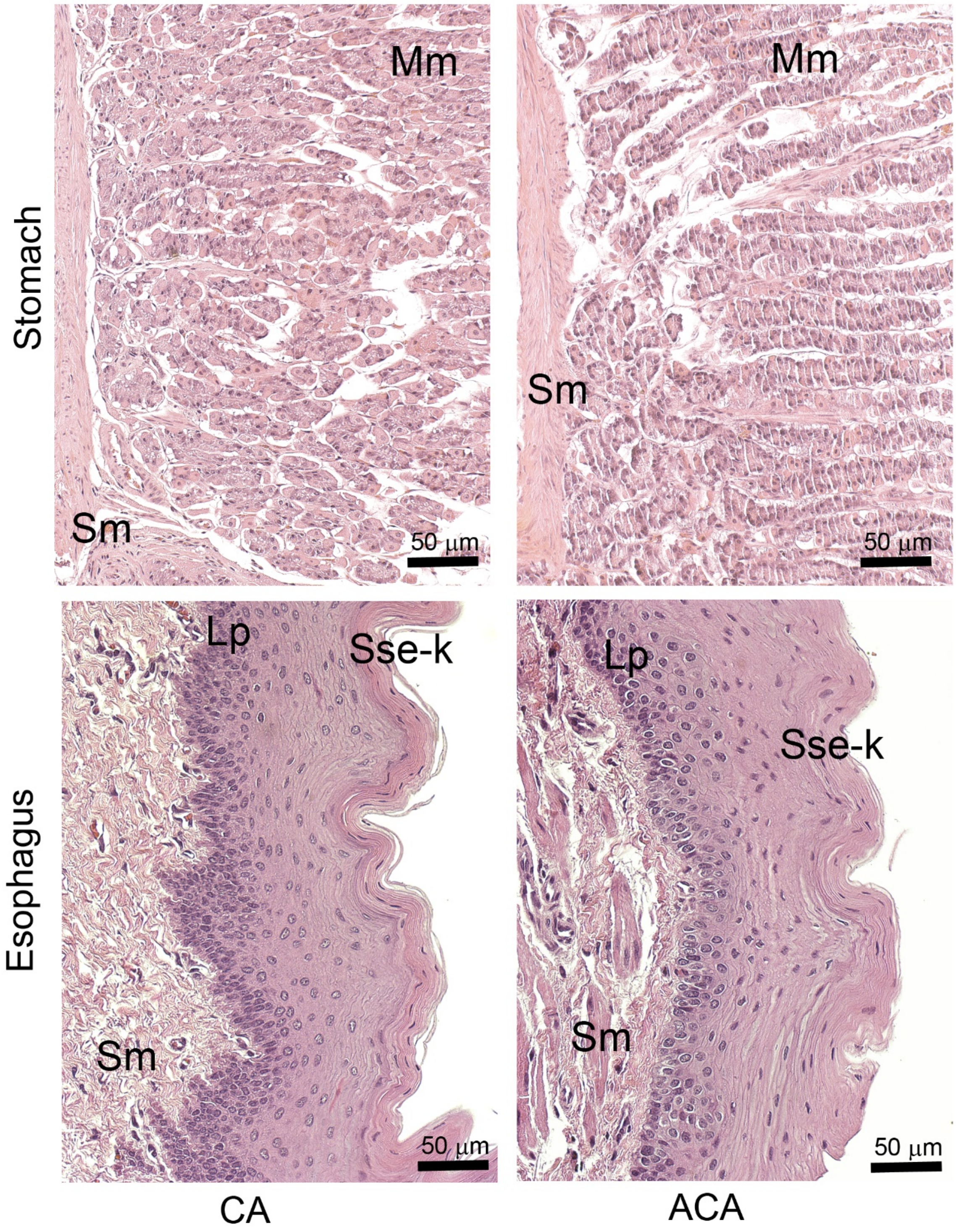
Stomach
Mm
Sm
50 µm
Mm
Sm
50 µm
Esophagus
Lp
Sse-k
Sm
50 µm
Lp
Sse-k
Sm
50 µm
CA
ACA

Figure S16

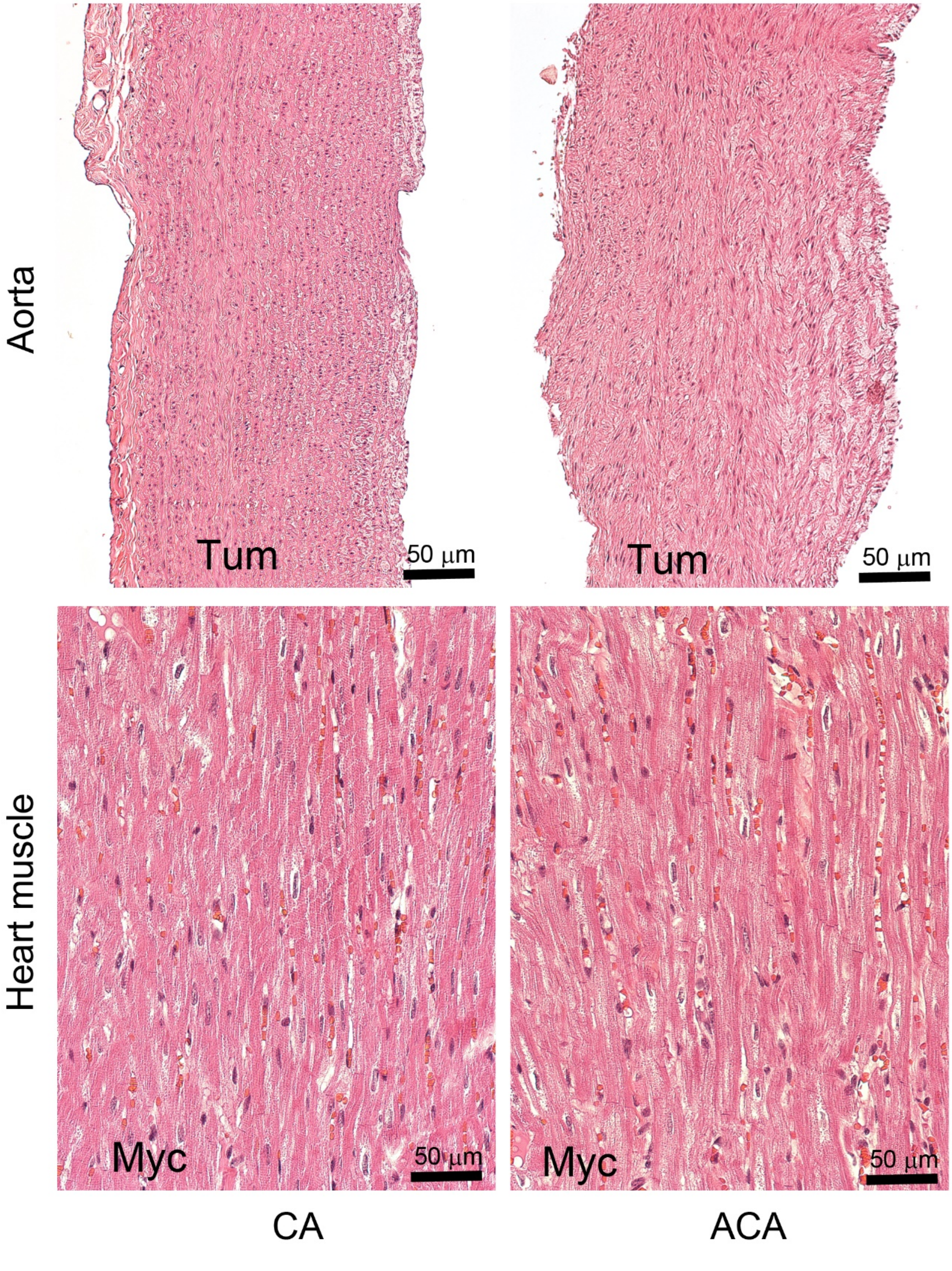

Figure S17

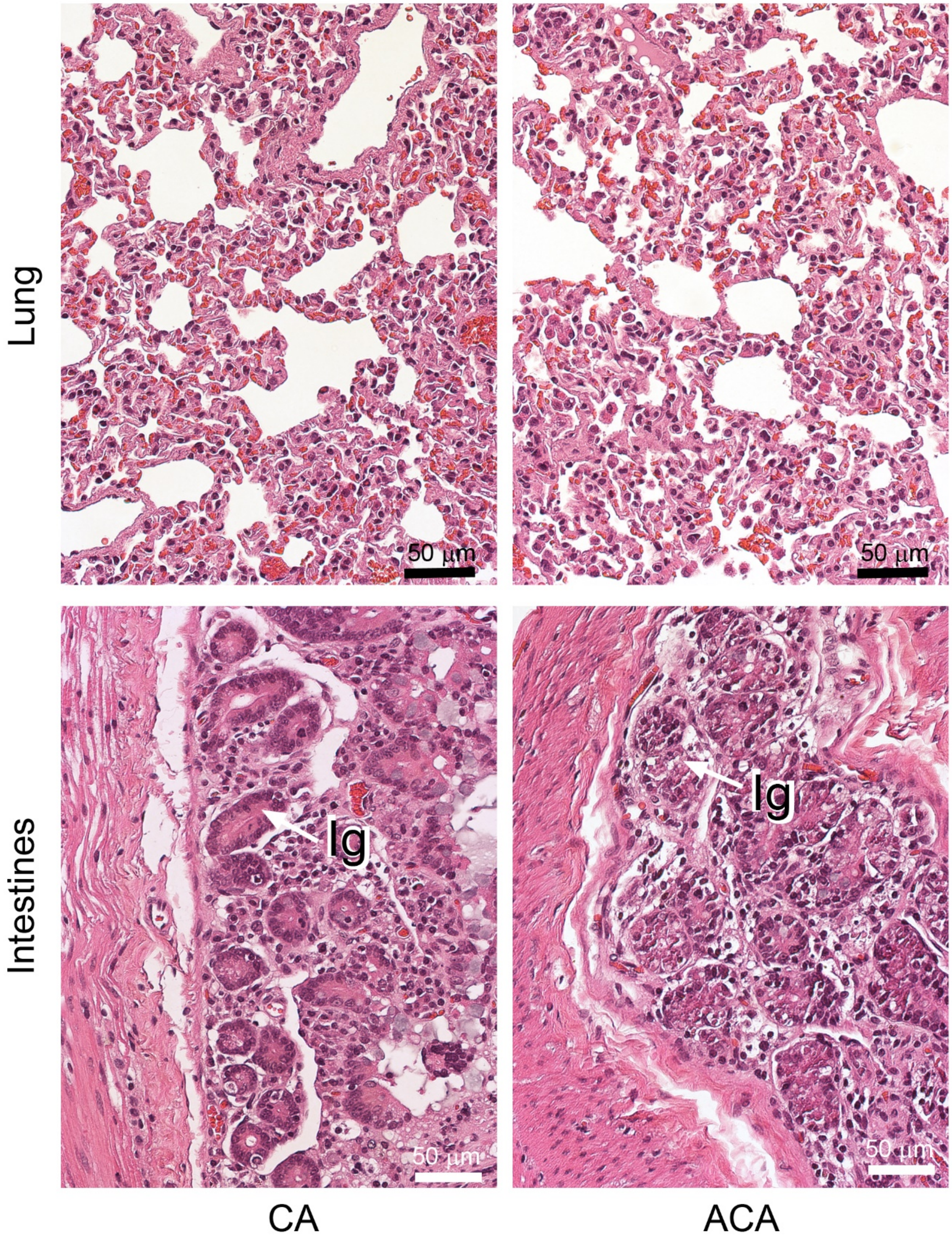

Figure S18

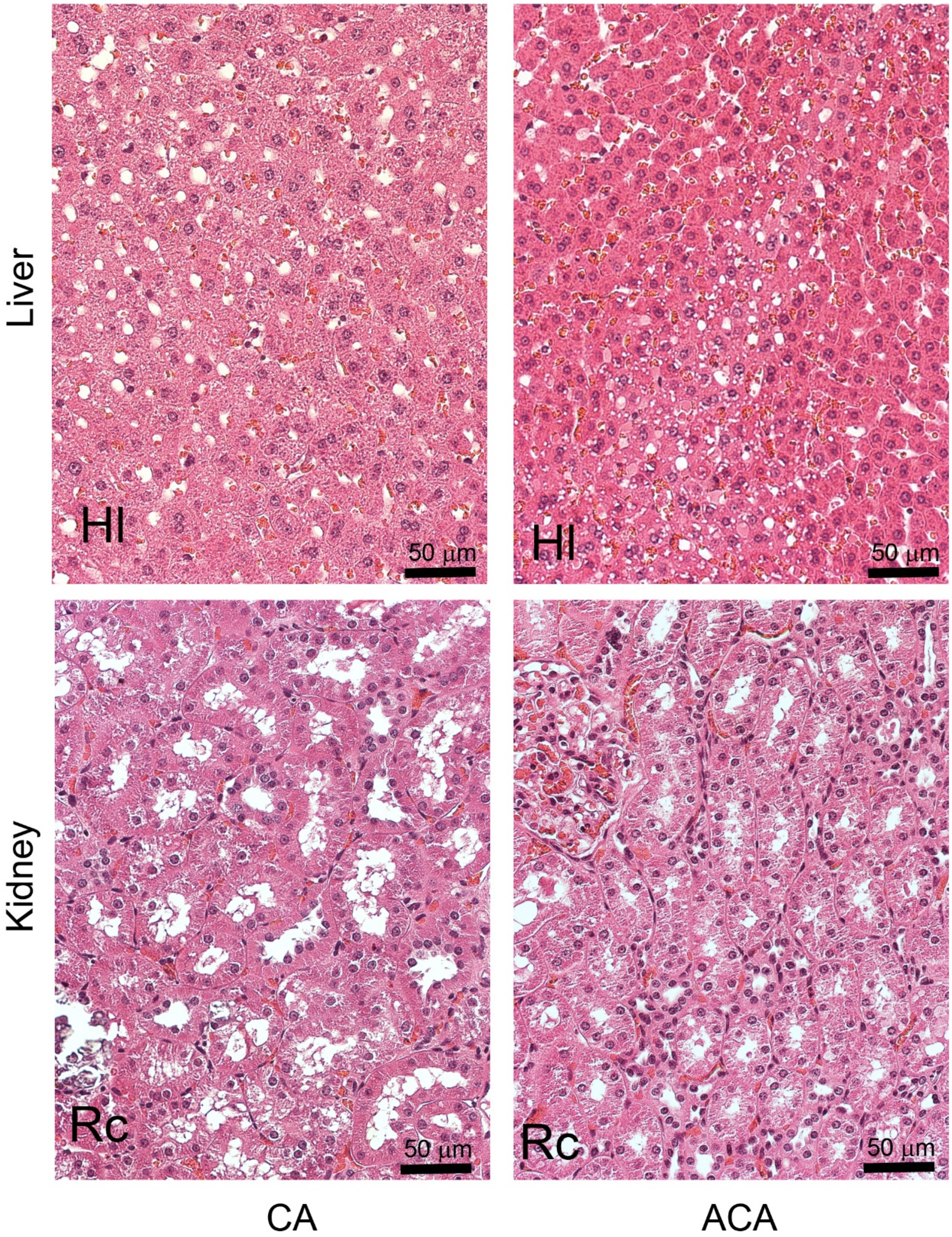

Figure S19

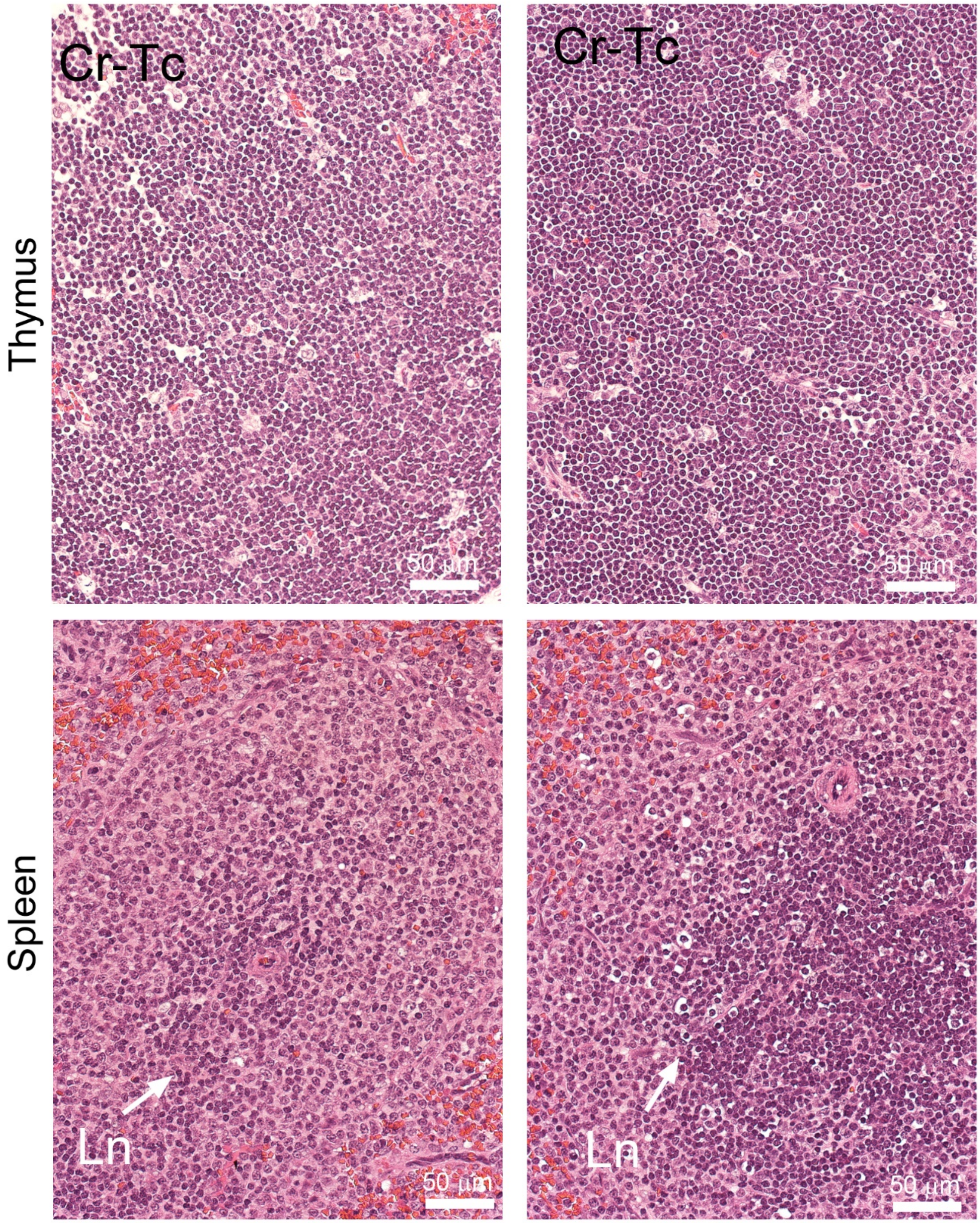

Figure S20

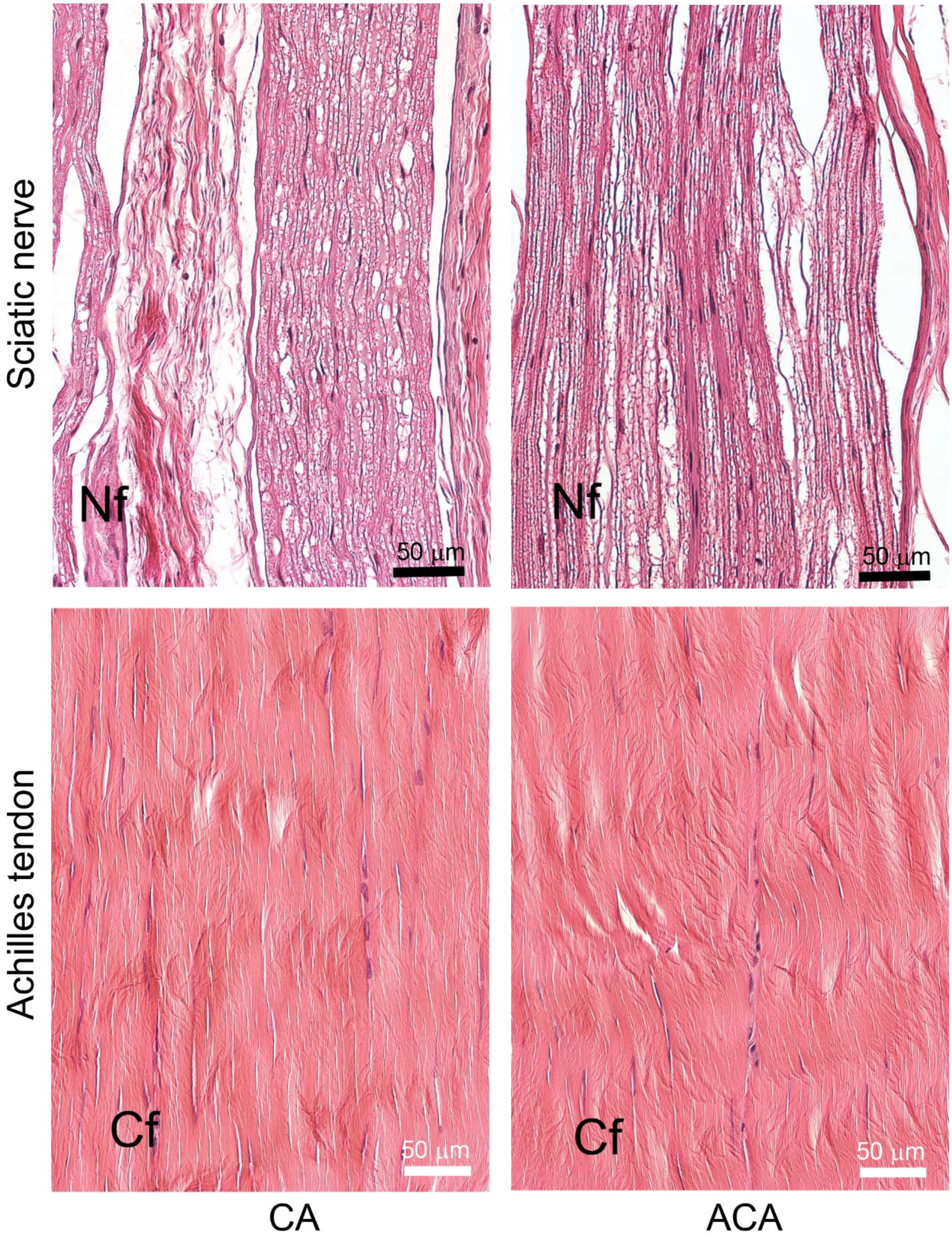
Sciatic nerve
Achilles tendon
Nf
Nf
Cf
Cf
50 μm
50 μm
50 μm
50 μm
CA
ACA

Figure S21

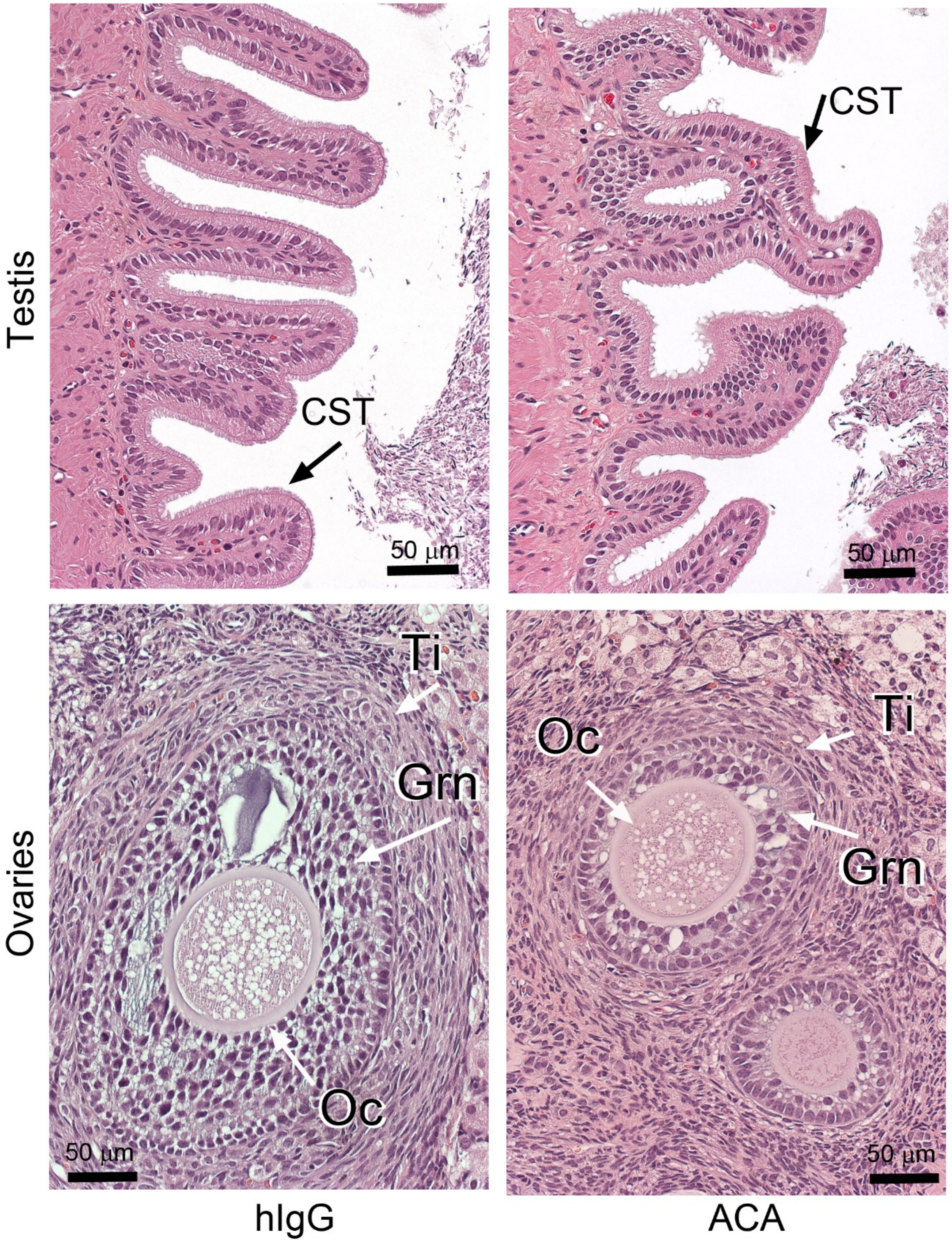
Testis
Ovaries
CST
CST
50 µm
50 µm
Ti
Grn
Oc
Oc
Ti
Grn
50 µm
50 µm
hIgG
ACA

Figure S22
